# Profile hidden Markov model sequence analysis can help remove putative pseudogenes from DNA barcoding and metabarcoding datasets

**DOI:** 10.1186/s12859-021-04180-x

**Published:** 2021-05-19

**Authors:** T. M. Porter, M. Hajibabaei

**Affiliations:** Department of Integrative Biology and Centre for Biodiversity Genomics, University of Guelph, 50 Stone Road East, Guelph, ON Canada

**Keywords:** Nuclear encoded mitochondrial sequences, Pseudogene, NuMT, Bioinformatics, COI mtDNA, DNA barcode, Metabarcode, Hidden Markov model

## Abstract

**Background:**

Pseudogenes are non-functional copies of protein coding genes that typically follow a different molecular evolutionary path as compared to functional genes. The inclusion of pseudogene sequences in DNA barcoding and metabarcoding analysis can lead to misleading results. None of the most widely used bioinformatic pipelines used to process marker gene (metabarcode) high throughput sequencing data specifically accounts for the presence of pseudogenes in protein-coding marker genes. The purpose of this study is to develop a method to screen for nuclear mitochondrial DNA segments (nuMTs) in large COI datasets. We do this by: (1) describing gene and nuMT characteristics from an artificial COI barcode dataset, (2) show the impact of two different pseudogene removal methods on perturbed community datasets with simulated nuMTs, and (3) incorporate a pseudogene filtering step in a bioinformatic pipeline that can be used to process Illumina paired-end COI metabarcode sequences. Open reading frame length and sequence bit scores from hidden Markov model (HMM) profile analysis were used to detect pseudogenes.

**Results:**

Our simulations showed that it was more difficult to identify nuMTs from shorter amplicon sequences such as those typically used in metabarcoding compared with full length DNA barcodes that are used in the construction of barcode libraries. It was also more difficult to identify nuMTs in datasets where there is a high percentage of nuMTs. Existing bioinformatic pipelines used to process metabarcode sequences already remove some nuMTs, especially in the rare sequence removal step, but the addition of a pseudogene filtering step can remove up to 5% of sequences even when other filtering steps are in place.

**Conclusions:**

Open reading frame length filtering alone or combined with hidden Markov model profile analysis can be used to effectively screen out apparent pseudogenes from large datasets. There is more to learn from COI nuMTs such as their frequency in DNA barcoding and metabarcoding studies, their taxonomic distribution, and evolution. Thus, we encourage the submission of verified COI nuMTs to public databases to facilitate future studies.

**Supplementary Information:**

The online version contains supplementary material available at 10.1186/s12859-021-04180-x.

## Background

The mitochondrial cytochrome c oxidase subunit 1 gene, COI, is the official animal barcode marker and large reference databases are available to help identify COI metabarcode sequences from soil, water, sediments, or mixed communities such as those collected from traps [1–3]. Crucially, the COI barcode marker is also a protein coding gene. This is in contrast with the ribosomal markers typically used for marker gene studies of prokaryotes or fungi [4–6]. Until recently, the methodology and bioinformatic pipelines for processing protein coding markers such as COI for animals, the maturase K gene (matK), or the ribulose bisphospate carboxylase large chain gene (rbcL) for plants have been treated in very much the same way, even using the same popular pipelines such as those used to process ribosomal RNA genes.

Pseudogenes are formed following a gene duplication event, where the duplicated region becomes non-functional but whose sequence still resembles the original gene sequence [7]. When a mitochondrial sequence has been inserted into the nuclear genome the result has been termed a nuclear mitochondrial DNA segment (nuMT) [8]. In this paper we use the term pseudogene in the general sense and the term nuMT specifically to refer to nuclear-encoded copies of mitochondrial DNA (mtDNA). The mechanism for this is uncertain but may involve the incorporation of mtDNA during the repair of chromosomal double strand breaks [9, 10]. Some nuMTs are ‘dead on arrival’ due to the different genetic code in the nuclear genome [11]. If the nuMT has only been recently introduced into the nuclear genome and only accumulated a few mutations, the sequence may closely resemble that of a functional COI gene with no frameshift or internal stop codons and may be referred to as a cryptic pseudogene [12]. More apparent pseudogenes may have been inserted into the nuclear genome in the past, followed by the divergence of the nuMT and mtDNA, each evolving at different rates and under different constraints [13]. In this case, the nuMT may exhibit stark changes in condon usage bias, transition:transversion ratios, GC content, decreased length, and have unexpected phylogenetic placement [14]. Since nuMTS have a slower rate of evolution than mtDNA, the primers used for PCR will bind to paralogous regions in nuMTs and will amplify nuMTS in addition to or even preferentially to the target mitochondrial sequence [13–17]. Inadvertently including pseudogenes in phylogenetic, biodiversity, or population analyses may introduce noise leading to overestimates of haplotype or species richness or misleading identifications or relationships [13, 16–23].

The methods needed to detect different types of pseudogenes will vary depending on whether or not many changes have accumulated. The obvious signs of non-functionality, frame shifts and stop codons, can lead to a truncated sequence. Less obvious signs of cryptic pseudogenes may be identified by examining raw Sanger chromatograms, similar to looking for evidence of heteroplasmy, by looking for double peaks [19]. The whole gene region may be examined looking for the presence of the control region and stop codon. Conserved regions such as in the inner mitochondrial membrane alpha helices can be examined for changes [24]. The rate of evolution in a COI mtDNA gene is faster than the rate of evolution of a translocated nuMT in the nuclear genome [15, 25]. This can be visualized in phylogenetic comparisons that include both mtDNA genes and nuMTs [17, 18, 20]. Paleonumts, apparent pseudogenes that integrated into the nucleus before mtDNA looks as it does now, can be identified by long branches that fail to group with orthologs [15, 26, 27]. Neonumts, on the other hand, have integrated into the nucleus more recently and still resemble ortholog sequences and cluster together on short branches [27]. Pseudogenes can also be identified using dN/dS ratios [28]. Pseudogenes are expected to have a similar rate of non-synonymous and synonymous substitutions for dN/dS ratios ~ 1. This is in contrast with a functional COI gene where substitutions tend to occur in non-synonymous sites so as to preserve amino acid composition and protein structure and dN/dS ratios are expected to be much less than 1. An alternative approach for pseudogene detection is hidden Markov model analysis. For example, a pseudogene detection method that uses tree-based HMMs was shown to identify pseudogenes better than a dN/dS approach [29, 30].

A hidden Markov model (HMM) can be used to describe features in groups of related biological sequences [31]. Non-technical reviews on HMMs and how they can be used to address biological problems are available [32, 33]. Briefly, in a multiple sequence alignment, residues can occur in a match, insertion, or deletion state. Each state is also associated with its own set of emission probabilities equivalent to the frequency of each residue in a column of the alignment. There are also transition probabilities associated with moving from one state to the next along the length of the alignment from the 5’ to 3’ end. The model generates two types of information: the hidden path through the model from state to state (a Markov chain) and the observed sequence (the residue emitted from each state). The probability of a path given an observed sequence and an HMM is calculated by taking the product of each transition and emission probability, or because these are really small numbers, summing the log probabilities. The best path through the model is the one with the highest probability.

Innovative methods for processing COI sequence data have arisen in recent years. For example, COI marker analysis need not be limited to operational taxonomic units (OTUs), but may also include the use of exact sequence variant (ESV) analysis for improved taxonomic resolution and permit intraspecific phylogeographic analyses [34–37]. Bioinformatic tools to remove sequence artefacts and noise specifically from COI datasets have also become available [38–40]. COI nuMTs have been discussed in the literature largely with regards to COI barcoding efforts [18, 19, 41] and only recently have tools appropriate for screening nuMTs from large batches of COI sequences become available [42]. The objective of this work is to develop methods to remove apparent pseudogenes from large datasets.

## Results

**Fig. 1:**
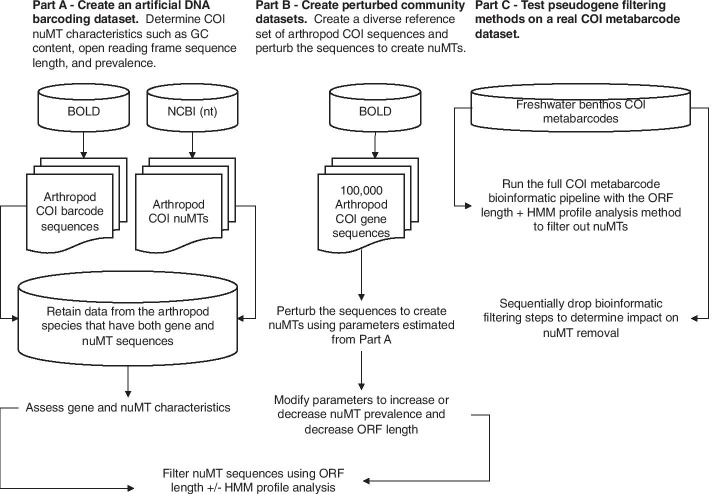
Overview of methods to determine COI nuMT characteristics and test methods for nuMT removal. Dataflow for our **a** artificial DNA barcode dataset, **b** perturbed community datasets, and **c** real freshwater COI metabarcode dataset. Abbreviations: BOLD = Barcode of Life Data System; COI = cytochrome c oxidase subunit I mtDNA gene; HMM = hidden Markov model; NCBI = National Centre for Biotechnology Information; nt = nucleotide; ORF = open reading frame

**Fig. 2:**
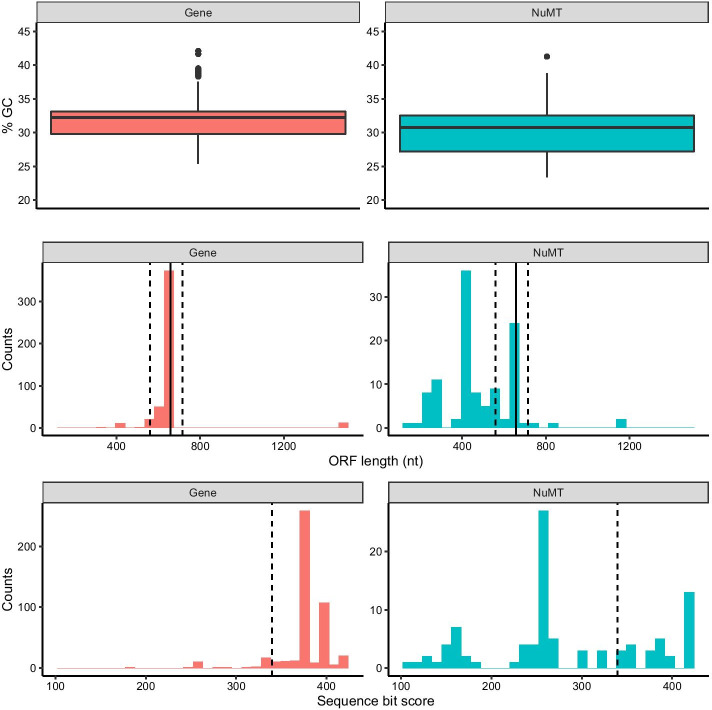
NuMTs tend to have lower GC content, shorter open reading frames, and smaller bit scores. Based on the artificial DNA barcoding dataset described in Table 1. The top panel shows GC content (%) in gene and nuMT sequences. The middle panel shows the sequence length distribution for the longest retained open reading frame. The solid vertical line indicates the length of a typical COI barcode at 658 bp. The two vertical dashed lines shows the boundaries for identifying ORFs with outlier lengths. The bottom panel shows the HMMER3 sequence bit score distribution. The vertical dashed line shows the cutoff for identifying outlier scores

Our artificial DNA barcode dataset that included 10 species with both gene and nuMT sequences allowed us to compare differences in GC content, length, and dN/dS ratios (Fig. 1). In Fig. 2, we show that COI nuMTs tend to have a slightly lower median GC content, shorter open reading frame (ORF) lengths, and shorter full sequence bit score values from HMM profile analyses. Additional file 1: Figure S1, shows how COI genes tend to accumulate substitutions in synonymous sites where a nucleotide changes does not result in the change of an amino acid; whereas COI nuMTs tend to accumulate substitutions in non-synonymous sites where a nucleotide change results in the change of an amino acid. After correcting for pairwise comparisons that could yield unreliable dN/dS ratios, where the number of substitutions at synonymous sites is < 0.01 or > 2, we were only able to calculate dN/dS ratios for COI gene sequences but not for nuMT sequences. Top BLAST hit analysis shows that all nuMTs had a top BLAST hit to another sequence from the expected species (92–100% identity). In some cases, the top BLAST match for a known nuMT was to another COI sequence annotated as a nuclear copy of a mitochondrial gene. More often, the top match for a nuMT was to a COI gene sequence. This indicates that in some cases, careful analysis of top BLAST hit output could help flag putative nuMTs. Additional file 1: Figures S2–S11, show COI phylograms for each species. In some cases, nuMTs form their own clusters (e.g., *Bemisia tabaci*, *Goneplax rhomboides*, *Melissotarsus insularis*), often on long branches (e.g., *Bemisia tabaci*, *Xylosandrus germanus*, *Triatoma dimidiate*, *Trialeurodes vaporariorum*, *Goneplax rhomboides*, *Ectatomma gibbum*), but occasionally nuMTs are found in clades intermixed with regular genes and little sequence divergence to distinguish them (e.g., *Melissotarsus insularis*, *Lepidocyrtus cyaneus*, *Halictus rubicundus*, *Cyphoderris monstrosa*). The proportion of nuMTs in these species that are putative paleonumts or neonumts is shown in Table 1.

**Table 1:** Summary of an artificial DNA barcoding dataset containing known arthropod COI nuMTs

Class	Order	Species [citation]	Gene sequences (% of total)	nuMT sequences (% paleonumts / % neonumts) (% of total)	Subtotals (% of total)
Insecta	Coleoptera	*Xylosandrus germanus* [98, 99]	33	1 (0/100)	34 (5.6)
Insecta	Hemiptera	*Bemisia tabaci* [100–103]	252	7	259 (43.7)
Insecta	Hemiptera	*Trialeurodes vaporariorum* [98, 103]	3	1 (0/100)	4 (0.7)
Insecta	Hemiptera	*Triatoma dimidiata* [104]	9	1 (0/100)	10 (1.7)
Insecta	Hymenoptera	*Ectatomma gibbum* [105]	6	1 (0/100)	7 (1.2)
Insecta	Hymenoptera	*Halictus rubicundus* [98, 106, 107]	29	2 (100/0)	31 (5.2)
Insecta	Hymenoptera	*Melissotarsus insularis* [108]	135	79 (61/39)	214 (36.1)
Insecta	Orthoptera	*Cyphoderris monstrosa* [27, 98]	7	14 (93/7)	21 (3.5)
Collembola	Entomobryomorpha	*Lepidocyrtus cyaneus*	5	1 (100/0)	6 (1.0)
Malacostraca	Decapoda	*Goneplax rhomboides* [109]	2	5 (20/80)	7 (1.2)
		Subtotals	481 (81)	112 (19)	593

Table 2 compares the sensitivity and specificity of two pseudogene removal methods on this dataset. Additional file 1: Figure S12, shows how we calculated sensitivity and specificity for each pseudogene removal method. Sensitivity refers to the true positive rate, in this case the number of pseudogenes correctly filtered out of the dataset. Specificity refers to the true negative rate, in this case, the number of gene sequences correctly retained. For our artificial DNA barcoding dataset including COI gene and nuMT sequences from 10 species, sensitivity (73%) is slightly higher for the ORFfinder + HMM profile analysis pseudogene removal method and the specificity is the same for each pseudogene removal method (90%).

**Table 2:** Sensitivity and specificity for two pseudogene filtering methods

Experiment	Dataset	Type of mutations	Sensitivity (%)		Specificity (%)	
			ORFfinder	ORFfinder + profile HMM analysis	ORFfinder	ORFfinder + profile HMM analysis
Artificial DNA barcoding dataset. COI genes and nuMTs from 10 species	Full length COI barcode and nuMT sequences	N/A	70	73	90	90
Perturbed community dataset	Full length COI barcode and simulated nuMTs	GC—> AT	31	27	99	~ 100
Perturbed community dataset	Full length COI barcode and simulated nuMTs	Frameshift	88	94	~ 100	~ 100
Perturbed community dataset	Short COI barcode and simulated nuMTs	GC—> AT	17**—50*	6**—15*	99	~ 100
Perturbed community dataset	Short COI barcode and simulated nuMTs	Frameshift	42**—58*	61**—87*	99	99*—~ 100**
Perturbed community dataset	Full length COI barcode and twice as many nuMTs	GC—> AT	17	0	99	~ 100
Perturbed community dataset	Full length COI barcode and twice as many nuMTs	Frameshift	0	0	~ 100	~ 100
Perturbed community dataset	Full length COI barcode and half as many nuMTs	GC—> AT	39	36	95	96
Perturbed community dataset	Full length COI barcode and half as many nuMTs	Frameshift	95	98	96	99

Sensitivity refers to the true positive rate, our ability to correctly identify known or simulated nuMTs. Specificity refers to the true negative rate, our ability to correctly identify COI genes. * 5’ fragment. ** 3’ fragment

**Fig. 3:**
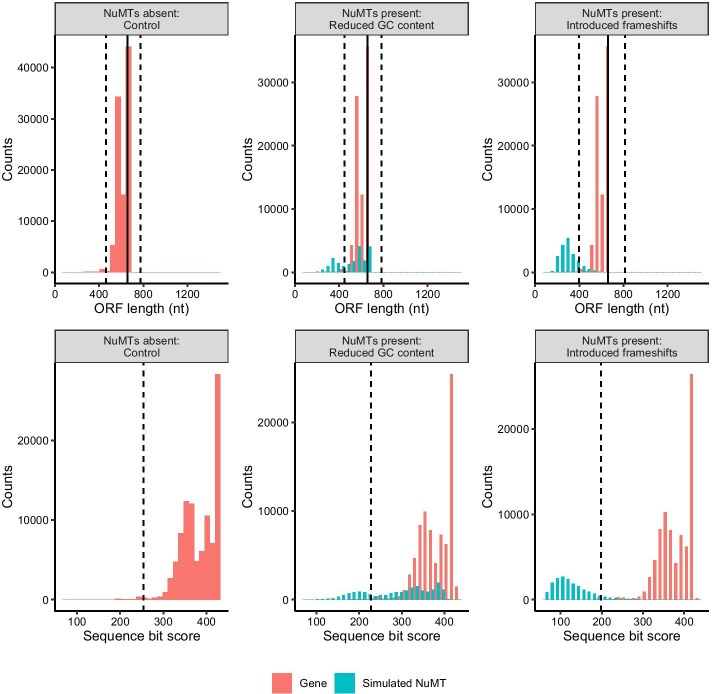
Reducing GC content and introducing frameshifts reduces ORF lengths and bit scores. Each column shows the results from a particular perturbed community dataset: a controlled community with nuMTs absent, a community with nuMTs that have a reduced GC content, and a community with nuMTs where we introduced frameshift mutations. The top panel shows the length variation of sequences in the longest retained open reading frame. The solid vertical line indicates the length of a typical COI barcode at 658 bp. The two vertical dashed lines shows the boundaries for identifying ORFs with outlier lengths. The bottom panel shows the HMMER3 sequence bit scores. The vertical dashed line shows the cutoff for identifying sequences with low outlier scores

We used our observations from the artificial DNA barcode dataset with COI genes and nuMTs from the same 10 species to guide the perturbation of community datasets comprised of 100,000 COI barcode sequences randomly sampled from the Barcode of Life Data System (BOLD) where we could manipulate parameters in different ways. In our perturbed community datasets of full length COI barcode sequences, we found that it was easier to filter out nuMTs caused by frameshift mutations (sensitivity 88–94%) rather than point mutations that reduced GC content (sensitivity 27–31%) (Fig. 3 and Table 2). As shown in Table 2, for full length COI barcode sequences, each nuMT removal method performed with similar specificity (99–100%).

We also analyzed additional perturbed community datasets by adjusting the length of the COI barcodes from full length to half length (~ 329 bp) as this is similar to the length of COI metabarcode sequences. As shown in Additional file 1: Figure S13, it is more difficult to filter out short nuMTs compared with full length COI barcodes. Table 2 shows that for half-length COI sequences, nuMT removal sensitivity is better for nuMTs generated by introducing frameshift mutations (42–87%) rather than with nuMTs where we reduced GC content by introducing point GC—>  AT mutations (6–50%). Sensitivity is also generally higher when removing nuMTs from the 5’ end of the COI barcode region (15–87%) compared with the 3’ end (6–61%). NuMT removal specificity is similar across pseudogene types and removal methods (99–100%).

Since we don’t really know how prevalent pseudogenes are in metabarcode datasets, we tested the effect of our pseudogene removal methods on a perturbed community dataset where there are many pseudogenes (38% instead of 19% in previous analyses). Additional file 1: Figure S14, shows that doubling the proportion of pseudogenes greatly reduces the number of simulated pseudogenes removed with either method. As shown in Table 2, pseudogene removal sensitivity is poor (0–17%) but specificity is high using either removal method (99–100%). Next, we ran the opposite analysis where there are few pseudogenes in the community (9.5% instead of 19% in previous analyses). Additional file 1: Figure S15, shows that reducing the number of pseudogenes in the community increases the number of simulated pseudogenes removed, especially when pseudogenes are caused by introducing frameshift mutations. As Table 2 shows, the sensitivity of pseudogene removal is high when pseudogenes are created by introducing frameshift mutations (95–98%), low when pseudogenes are created by reducing GC content through GC—> AT point mutations (36–39%), and the specificity is high for either type of pseudogene or removal method (95–99%).

**Fig. 4:**
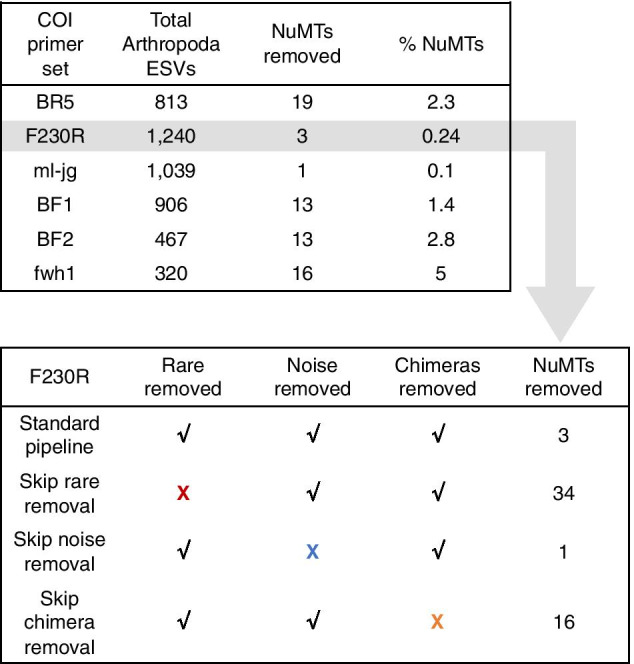
Removing rare sequences also removes apparent pseudogenes. The number of removed putative nuMTs was calculated for each of the 5 amplicons from a freshwater COI metabarcode dataset. Note, that we only compared results across Arthropoda ESVs. Using the SCVUC v4.3.0 bioinformatic pipeline, the F230R amplicon recovered the greatest ESV richness (top box) so we used this as a test case for further tests (bottom box). To determine whether current bioinformatic processing steps already help to remove apparent pseudogenes, we dropped one step at a time: removal of rare sequences, removal of noisy sequences, and removal of chimeric sequences

Because the ORFfinder + HMM profile analysis method for removing pseudogenes had the highest sensitivity for short COI sequences when nuMTs were simulated by introducing frameshift mutations, we used this method to test our ability to remove nuMTs with a real COI metabarcode dataset. Note that analyses were limited to only arthropod ESVs because most of the primer sets in the study were designed to specifically target this group (Additional file 1: Table S1). As shown in Fig. 4, the total number of arthropod ESVs was highest for the F230R amplicon (1240) and least for the fwh1 amplicon (320). The greatest number of nuMTs was detected and removed from the BR5 amplicon (19) and least for the ml-jg amplicon (1). Overall, the greatest percentage of nuMTs out of all ESVs was detected from the fwh1 amplicon (5%) and least for the ml-jg amplicon (0.1%). Because the F230R amplicon detected the greatest ESV richness, we used this amplicon to determine how existing bioinformatic processing steps affects nuMT removal. Using the SCVUC v4.3.0 metabarcode pipeline with ORFfinder + HMM profile analysis pseudogene removal, three F230R nuMTs were removed from the dataset. Omitting the rare sequence removal step from the bioinformatic pipeline resulted in the largest number of pseudogenes detected, 34. Omitting the denoising step results in 1 pseudogene detected and may reflect a situation where there are so many pseudogenes in the dataset that it becomes difficult for our method to detect them. Omitting the chimera removal step results in 16 pseudogenes removed. This suggests to us that at least some apparent pseudogenes are probably already being removed during regular bioinformatic processing, especially during the rare sequence removal step as we would expect from the literature [43–47].

## Discussion

Are all the COI sequences filtered out using ORFfinder + HMM profile analysis nuMTS? This method of pseudogene removal cannot distinguish between genuine pseudogenes and technical issues involving PCR or sequencing that cause frameshifts and the introduction of premature stop codons. It is possible that even after bioinformatic processing such as denoising, chimera removal and rare sequence removal, artefactual sequences may be missed and subsequently removed with these pseudogene removal methods. Although it is possible that genuine COI sequences could be removed using these methods, the specificity for pseudogenes is high (96–100%) and the number of COI gene sequences removed is very low in our artificial DNA barcode and perturbed community datasets.

There are biological reasons why genuine mitochondrial sequences may be misclassified as pseudogenes. For example, in bivalves, male and female lineages of mitochondria may lead to fully functional gene copies with divergent sequences [41, 48, 49]. Though this type of sequence could complicate COI barcoding or phylogenetic analysis, this would not be filtered out by our methods because as functional COI genes they should produce a good bitscore during profile HMM analysis. There are also cases in the literature where as a cell ages oxidative stress damages DNA that is then repaired by enzymes with reduced activity [41, 50]. Unrepaired mutations including deletions, duplications, and point mutations can accumulate in aging cells. Since truncated mtDNA can be replicated faster than full length mtDNA, it is possible for partially deleted mtDNA to accumulate [51]. Similarly, damaged DNA caused by poor preservation could cause COI sequences with frameshifts or premature stop codons to look like nuMTs.

How can pseudogenes be avoided? Indicators for the presence of pseudogenes include extra bands after PCR, sequence ambiguities when comparing both strands, frameshift mutations, premature stop codons, and unexpected phylogenetic position [14]. Strategies for avoiding nuMTs in single specimens may include using muscle tissue for DNA extraction as it is naturally enriched with mtDNA, purifying mitochondria before DNA extraction, by amplifying long stretches of mtDNA with PCR, or targeting RNA using reverse transcription PCR [14, 18]. Even when working with environmental DNA samples it is possible to apply some of these techniques to avoid nuMTs. For example, mitochondrial enrichment from homogenized tissues is possible and could be applied to freshwater benthic collections or insects collected from traps [52]. Additionally, long range PCR targeting mitochondrial DNA from water samples can allow for the construction of whole mitogenomes from fish [53]. Environmental RNA has also been used to detect microbes by targeting ribosomal RNA or using messenger RNA to target COI [54–58]. For large scale studies, however, introducing additional steps such as mitochondrial purification or reverse transcription could be costly and time consuming and a bioinformatic method to handle pseudogenes would be useful.

Our results show that our ability to detect nuMTs is hindered by short COI metabarcodes or if the abundance of sequenced pseudogenes is very high. On the other hand, we also show that in a freshwater benthos COI metabarcode dataset we can remove up to 5% of arthropod ESVs as putative nuMTs even when other filtering steps are in place. It is quite possible that additional nuMTs remain in the dataset, undetected by our pipeline. Our pseudogene removal methods may not be able to remove cryptic pseudogenes, but these may still be useful for making higher level taxonomic assignments, though they may inflate richness at the species or haplotype level. Failure to remove low quality and artefactual sequences can result in inflated richness estimates in biodiversity studies, as has been shown for grashoppers and crayfish [18]. Pseudogenes are unlikely to affect community composition or beta diversity analyses if they are rare in the dataset as these analyses are less likely to be affected by the presence of rare sequences.

The use of phylogenetic based methods is common in COI barcoding studies where the presence of nuMTs can be problematic [17, 18, 21, 23]. For example, a study of the great apes, showed that nuMTS are commonly sequenced in gorillas and complicate phylogenetic analyses [59]. It has also been suggested that pseudogenes are common in *Drosophila melanogaster* and in fish where they were once thought to be absent [60, 61]. There is a positive correlation between nuclear genome size and abundance of nuMTS [10]. This is especially important in arthropods, for the order Orthoptera, a group of grasshoppers, locusts, crickets, and katydids known to have very large genomes [27]. In our study, we observed a spectrum of branching patterns between nuMTs and orthologs. Apparent pseudogenes, also referred to as paleonumts in the literature, were found on long branches and likely represent a nuclear insertion event in the past followed by the independent evolution of nuMT and mtDNA [27]. At the other end of the spectrum, cryptic pseudogenes, also referred to as neonumts, were likely of more recent origin in the nuclear genome, clustering with COI gene sequences on short branches [27]. The signatures of both neonumts and paleonumts can be found in the same species (Table 1).

The increasing use of COI metabarcodes for intraspecific analyses using ESVs could also be impacted by the presence of cryptic pseudogenes. In avian and insect studies, nuMTs have complicated population genetic studies and there have been calls for careful screening of sequences prior to launching large scale population level analyses using mitochondrial markers [12, 13, 22]. In some cases, the pseudogene sequences are highly conserved, and the length of gene and pseudogene sequences are the same after PCR [22]. Sequence differences due to heteroplasmy or nuMTs could be distinguished by isolating mtDNA and nuclear DNA separately from single individuals [22]. The use of ORFfinder + HMM profile analysis, screening out hits with low outlier sequence bit scores, could be used as a first pass method for removing apparent pseudogenes. An automated method such as what we use in the SCVUC metabarcode pipeline in this study is more straight-forward to score compared with trying to identify pseudogenes from phylogenies by eye as branching patterns between genes and pseudogenes are not always clear cut. To detect cryptic pseudogenes careful analysis of species level sequence alignments should still be carried out, for instance, to check for low GC content, high dN/dS ratios, and codon usage bias.

Hidden Markov model profile analysis is not a commonly used method to analyze COI metabarcodes on its own, but it is used under the hood for many other applications. Perhaps the most well-known example is as a part of the BOLD identification engine that can be used to identify unknown barcode sequences [2]. The ITSx extractor is a program used to process fungal ITS metabarcodes by identifying and removing the conserved gene regions adjacent to the internal transcribed spacer regions (ITS1 and ITS2) [62]. HMMs are already used in the Pfam database of protein families [63]. HMM analysis is also used to place 16S rRNA gene sequences in a reference phylogeny in PICRUST2 [64]. We have also made available a multi-marker metabarcode snakemake pipeline that processes paired-end Illumina reads that provides a pseudogene filtering step for protein coding markers called MetaWorks that can be found at https://github.com/terrimporter/MetaWorks. Furthermore, though our current work has focused on arthropod sequences, taxon-specific HMM profiles could be developed for additional macroinvertebrate groups of interest for biomonitoring such as Tubellaria, Gastropoda, Bivalvia, Polychaeta, Oligochaeta, and Hirudinea to permit more refined HMM-profile analyses [65]. It would also be useful to develop HMM profiles for other commonly used protein coding markers such as rbcL and matK to facilitate nuMT removal from large plant sequence datasets.

## Conclusions

We have shown that it is possible to screen out apparent pseudogenes using ORF length filtering alone or combined with HMM profile analysis for greater sensitivity when pseudogene sequences contain frameshift mutations. Our pseudogene removal approach was most effective on datasets of the full length COI barcode sequence region but is less effective for shorter sequences (~ 300 bp). Now that newer sequencing technologies such as LoopSeq, compatible with Illumina sequencing platforms but currently only available for RNA genes, or HiFi circular consensus sequencing (PacBio), it may one day be possible for COI metabarcoding to target the full length of the barcoding region to facilitate more efficient nuMT detection [39, 66–68]. It would also be helpful if DNA barcode studies reported and deposited full length verified pseudogenes into public databases when possible. Having key words such as ‘nuclear copy of mitochondrial gene’ or ‘pseudogene’ in the description would be essential to quickly flag hits to such sequences. As the analysis of metabarcode sequences from protein-coding genes shifts towards the use of ESVs, it is more important than ever to reduce noise by removing pseudogenes to avoid inflated richness estimates or misleading phylogenetic or population level analyses. In this study we identified the need for a pseudogene filtering step in bioinformatic pipelines used to process protein-coding genes and we hope this work illustrates why this is needed and how it can be implemented.

## Methods

We used three approaches in this study: A) We created an artificial DNA barcode dataset by compiling a set of annotated COI genes and nuMTs from BOLD and the National Center for Biotechnology Information (NCBI) nucleotide (nt) database for the same set of 10 species; B) We created perturbed COI community datasets by mining sequences from BOLD and simulating nuMTs, and C) We tested a pseudogene filtering method on a previously published freshwater benthos COI metabarcode dataset (Fig. 1).

### Part A: Creating an artificial DNA barcoding dataset

To create an artificial DNA barcode dataset where multiple sequences are generated for the same species, we retrieved high quality sequences from BOLD and known nuMTs mined from the NCBI nucleotide database for the same set of species. Sequences from the BOLD data releases were obtained from http://v3.boldsystems.org/index.php/datarelease. Nucleotide sequences for arthropods were selected, ensuring that there were no ambiguities in the nucleotide sequences. If either the nucleotide sequence or amino acid sequence were missing, then the record was discarded. A FASTA file containing arthropod COI nuMTs was obtained from the NCBI nucleotide database using an Ebot script with the search term “Arthropoda[ORGN] AND pseudogene[TITL] AND (COI[GENE] OR CO1[GENE] OR coxI[GENE] OR cox1[GENE]) AND 50:2000[SLEN]” [69]. A few records had to be edited by hand to isolate the sequence region associated with the COI nuMT. We retrieved 481 COI nucleotide sequences from BOLD and 112 COI nuMT nucleotide sequences from the NCBI nucleotide database from the same 10 species (Table 1). We also indicated the percentage of nuMTs that we would classify as paleonumts with relatively long branch lengths or neonumts with relatively short branch lengths based on a neighbor joining analysis (described below). This dataset is further described in Additional file 1: Table S2 showing proportion of nuMTs, average length, and average GC content. 

GC content for COI gene and nuMT sequences were assessed in R v4.0.3 using the ‘seqinr’ package in RStudio v1.3.1093 [70–72]. We pooled all the sequences together, then proceeded to filter out just the nuMTs using two different methods:

The first method we used to remove pseudogenes involved screening out sequences with outlier open reading frame lengths that were very short or very long. This was done by translating arthropod ESVs using ORFfinder v0.4.3 into every possible open reading frame on the plus strand using the mitochondrial invertebrate genetic code, ignoring nested ORFs, and setting the minimum length to 30. The longest ORFs were retained. Outliers, putative pseudogenes or genuine sequences with PCR/sequencing errors, were identified as sequences shorter than the 25th percentile ORF length—(1.5 * interquartile length) and longer than the 75th percentile ORF length + (1.5 * interquartile length).

The second method we used to remove pseudogenes involved profile hidden Markov model (HMM) analysis. We compared each of our query sequences (comprised of COI genes and nuMTs translated into amino acid sequences) sequentially against an amino acid COI gene HMM profile representing 6162 arthropod barcode sequences. This was done by creating a profile HMM based on BOLD arthropod barcode sequences using HMMER v3.3 available from http://hmmer.org. HMMER3 is now nearly as fast as BLAST for protein searches [73]. The first step was building the COI barcode HMM profile: From the BOLD data releases iBOL phase 0.50 to 6.50, we retrieved all arthropod barcodes 600–700 bp in length. We sorted these sequences by decreasing length using the ‘sortbylength’ command in VSEARCH. We reduced the dataset size by clustering by 80% sequence similarity using the ‘cluster_size’ command and retaining the centroids sequences. As described above, arthropod ESVs were translated, and the longest ORFs and amino acid sequences were retained. The amino acid sequences were aligned with MAFFT v7.455 using the ‘auto’ setting [74]. The ORFs were also mapped to the amino acid alignment using TRANALIGN (EMBOSS v6.6.0.0) specifying the invertebrate mitochondrial genetic code [75]. The FASTA file comprised of 6162 amino acid sequences was converted to Stockholm format. This reference alignment was turned into a model that describes the probabilities for travelling a path along the length of the alignment that moves through match, insert, or deletion states. HMMER was used to build this nucleotide arthropod COI profile hidden Markov model (HMM) using the ‘hmmbuild’ command. The HMM was indexed using the ‘hmmpress’ command. The second step was to compare query sequences against the HMM profile: Individual arthropod amino acid sequences were then compared with the profile HMM using the ‘hmmscan’ command. One of the hmmscan outputs is a log odds ratio score (bit score) that compares the likelihood of the query sequence given the model to the likelihood of the query sequence given a random sequence model. Recall that our model is based on the COI barcoding region, so when a COI gene is used as the query we expected a high bit score and when a COI nuMT is used as the query we expected a low bit score. In this way, putative pseudogenes were identified if they had low outlier HMMER scores.

We also calculated the number of substitutions per non-synonymous and synonymous site. Gene sequences and pseudogene sequences were analyzed separately as follows: Amino acid sequences were aligned using MAFFT v7.455 using the ‘auto’ setting. A codon alignment was created using TRANALIGN (EMBOSS) by mapping the ORFs to the amino acid alignment using the invertebrate mitochondrial genetic code. We used the package ‘ggplot2’ to create all plots [76]. We used the ‘seqinr’ function ‘kaks’ to calculate the number of substitutions for non-synonymous and synonymous sites [70, 77]. Before calculating dN/dS ratios, we excluded pairwise sequence comparisons where the number of substitutions per synonymous site was < 0.01 (sequences too similar to yield reliable dN/dS) or > 2 (too many substitutions, near saturation, to yield a reliable dN/dS).

To assess how pseudogene sequences could be (mis)identified using the top BLAST hit method, we used the Megablast algorithm to find the most similar sequence in the NCBI nucleotide sequence database [78]. We used this method to verify that the expected species was a top match (skipping over the top match if it was the same as the query sequence or if it was an obvious contaminant) and whether or not the top match was to a gene or pseudogene sequence in the reference database. To further visualize phylogenetic divergence between gene and pseudogene sequences for each species, we aligned nucleotide sequences with MAFFT using the ‘auto’ setting. We used the neighbor joining (NJ) method of phylogenetic tree construction as it has been shown that NJ performs as well as, or in some scenarios even better than, maximum likelihood for discriminating among recently separated taxa [79]. The ‘fdnadist’ Phylip method in the EMBOSS package was used to calculate distances using the Kimura 2-parameter (K2P) model of nucleotide sequence evolution, the conventional approach used in the field of DNA barcoding [80, 81]. A neighbor joining tree was saved in Newick format using the ‘fneighbor’ Phylip method in EMBOSS. Statistical support at nodes was calculated by bootstrapping the multiple sequence alignment 1000 times using the ‘fseqboot’ Phylip method in the EMBOSS package then K2P distances and neighbor joining trees were constructed as described above. A majority rule consensus tree was constructed using the Phylip program ‘consense’ [81]. Bootstrap values from the consensus tree were mapped to the phylogram using TreeGraph2 v2.15.0-887 [82]. The tree was mid-point rooted and nodes rotated or collapsed where necessary to improve readability using FigTree v1.4.4 available from http://tree.bio.ed.ac.uk/software/figtree/. Further minor editing to improve readability was performed using Inkscape v1.0.1 available from https://inkscape.org/.

### Part B: Creating perturbed community sequence datasets

To test our pseudogene filtering methods on a more taxonomically diverse community of arthropods, we created perturbed community sequence datasets. We created an arthropod COI community based on 100,000 sequences randomly sampled from the BOLD data releases. We manipulated this community in different ways described below. In our first perturbed dataset, based on our simulated DNA barcoding results from Part A where ~ 19% of our dataset represented nuMTs, we introduced GC—> AT point mutations into 19% of the BOLD sequences. Also based on the results from Part A, we reduced the GC content in our simulated pseudogenes by 2.5%. In our second perturbed dataset, we inserted or deleted a base to introduce frameshift mutations and premature stop codons. To keep the rate of pseudogenization the same as the first artificial community, we introduced frameshift mutations, i.e. indels, in 2.5% of the bases in our simulated nuMTs. In the third perturbed dataset, we split COI barcode sequences in half to test whether our pseudogene filtering approach would work on shorter barcode sequences similar in length to those generated in COI metabarcoding studies (~ 300 bp). In a fourth perturbed dataset, we doubled the proportion of nuMTs in the mock community from 19 to 38%. In the fifth perturbed dataset, we halved the proportion of nuMTs in the mock community from 19% to 9.5%. Each of these datasets is further described in Additional file 1: Table S1 showing the proportion of pseudogenes in the dataset, average length, and average GC content.

### Part C: Testing pseudogene filtering methods using a COI metabarcode dataset

We used a previously published freshwater benthos COI metabarcode dataset to test our bioinformatic pipeline and two different pseudogene removal strategies [83]. We chose this dataset because it includes results from six different COI amplicons (BR5 [B, ArR5] ~ 310 bp, F230R [LCO1490, 230_R] ~ 229 bp, ml-jg [mlCOIintF, jgHCO2198] ~ 313 bp, BF1 [BF1, BR2] ~ 316 bp, BF2 [BF2, BR2] ~ 421 bp, fwh1 [fwhF1, fwhR1] ~ 178 bp) currently used in a variety of labs in the freshwater COI metabarcode literature [65, 84–90]. The primers and their target taxa are listed in Additional file 1: Table S2. Each amplicon covers sites across the COI barcoding region and the mode length ranges from 178 bp (fwh1) to 421 bp (BF2), averaging ~ 300 bp. The F230R and fwh1 amplicons align to the 5’ end of the barcoding region and the BR5, ml-jg, BF1, and BF2 amplicons align to the 3’ end of the barcode region.

**Fig. 5:**
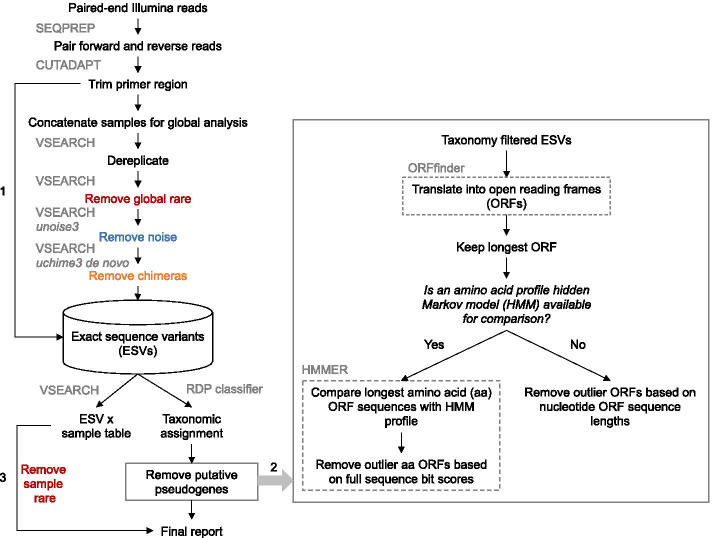
Overview of the metabarcoding bioinformatic pipeline that removes apparent pseudogenes. The SCVUC pipelines begins with Illumina paired-end reads. Arrow 1 indicates where primer trimmed reads are mapped to denoised exact sequence variants (ESVs) to create a sample x ESV table that contains read counts. Arrow 2 indicates where pseudogenes can be removed using two different approaches. The first method translates ESVs, retains the longest nucleotide open reading frame (ORF), then removes sequences with very small or very large outlier lengths. The second method retains the amino acid sequence from the longest ORF, does a profile HMM analysis, then removes sequences with very small outlier full sequence bit scores. Arrow 3 indicates where rare sequence clusters from each sample are removed and read numbers are mapped to the final report. The final report contains all ESVs for each sample, read numbers, ORF sequences, and taxonomic assignments with bootstrap support values

The COI metabarcoding bioinformatic pipelines SCVUC v4.1.0 and SCVUC v4.3.0 were used to process Illumina paired-end reads to output a set of taxonomically assigned ESVs (available from GitHub at https://github.com/Hajibabaei-Lab/SCVUC_COI_metabarcode_pipeline) (Fig. 5). SCVUC v4.1.0 removes putative pseudogenes using the ORFfinder method described above. SCVUC v4.3.0 removes putative pseudogenes using the ORFfinder + HMM profile analysis described above. This pipeline runs in a conda environment with a snakemake pipeline. Conda is an environment and package manager [91]. It allows most programs and their dependencies to be installed easily and shared with others. Snakemake is a python-based workflow manager [92]. The snakefile contains the commands needed to run a bioinformatic pipeline. The configuration file allows users to adjust parameter settings.

Raw paired-end reads were merged using SEQPREP v1.3.2 [93]. We set a minimum Phred quality score of 20 in the overlap region and a minimum 25 bp overlap. Primers were trimmed in two steps using CUTADAPT v2.6 setting a Phred quality score of 20 at the ends to count matches/mismatches, no more than 3 Ns allowed, and trimmed reads of at least 150 bp [94]. Sequence files were combined for a global analysis. Reads were dereplicated using VSEARCH v2.14.1 [95]. Denoised exact sequence variants (ESVs) were also generated using VSEARCH using the unoise3 algorithm [43]. This step clustered reads by 100% sequence identity, removed sequences with predicted errors, and globally rare sequences. Here we define rare sequences as clusters containing only one or two sequences. Putative chimeric sequences were removed using the uchime3_denovo algorithm in VSEARCH [44]. Denoised ORFs (ESVs) were taxonomically assigned using a naive Bayesian classifier trained with a COI reference set comprised of sequences mined from GenBank and the BOLD data releases [96, 97]. Rare sequences clusters were also removed from each sample. We then modified the pipeline to skip over several steps, one at a time, to see how this would affect the removal of apparent pseudogenes using the ORFfinder + profile HMM method: rare sequence removal, noise removal, chimeric sequence removal.

## Supplementary Information

**Additional file 1**. Includes supplementary Tables S1–S2 and supplementary Figures S1–S15.

## Data Availability

Key scripts and infiles used create simulated datasets are available from GitHub at https://github.com/terrimporter/PorterHajibabaei2021_pseudogene. The SCVUC COI metabarcode pipelines used in this study is also available on GitHub from https://github.com/Hajibabaei-Lab/SCVUC_COI_metabarcode_pipeline.

## Abbreviations

BLAST: Basic local alignment search tool
BOLD: Barcode of Life Data System
COI: Cytochrome c oxidase subunit 1 gene
dN/dS: Ratio of non-synonymous to synonymous substitutions
ESV: Exact sequence variant
GC content: Guanine-cytosine content
HMM: Hidden Markov Model
ITS: Internal transcribed spacer region in the ribosomal RNA operon
K2P: Kimura 2-parameter model of nucleotide substitution
matK: Maturase K gene
mtDNA: Mitochondrial DNA
nuMT: Nuclear encoded mitochondrial sequence
NCBI: National Center for Biotechnology Information
ORF: Open reading frame
OTU: Operational taxonomic unit
rbcL: Ribulose bisphosphate carboxylate large chain gene
