# Supplementary material for: Profile hidden Markov model sequence analysis can help remove putative pseudogenes from DNA barcoding and metabarcoding datasets
Source: BMC Bioinformatics. 2021 May 19;22:256. doi: 10.1186/s12859-021-04180-x (PMC8136176; doi:10.1186/s12859-021-04180-x)
Supplement: Supplementary file 1 — Additional file 1. Includes supplementary Tables S1–S2 and supplementary Figures S1–S15. [file 12859_2021_4180_MOESM1_ESM.pdf]

Profile hidden Markov model sequence analysis can help remove putative pseudogenes from DNA barcoding and metabarcoding datasets

Porter, T. M., Hajibabaei, M.

Supplementary Material

Table S1. Primers used in the freshwater benthos COI metabarcode dataset used in Part C (Hajibabaei et al., 2019 PLoS ONE).

Amplicon	Primer	Target	Primer sequence (5'-3')	Reference
BR5	B	Freshwater benthic macroinvertebrates	CCIGAYATRGCITTYCCICG	(Hajibabaei, Spall, Shokralla, & van Konynenburg, 2012)
	ArR5	Tropical arthropods	GTRATIGCICCIGCIARIACIGG	(Gibson et al., 2014)*
F230R	LCO1490	Metazoan macroinvertebrates	GGTCAACAAATCATAAAGATATTGG	(Folmer, Black, Hoeh, Lutz, & Vrijenhoek, 1994)
	230_R	Arthropods	CTTATRTTRTTTATICGIGGRAAIGC	(Gibson et al., 2015)
ml-jg	mlCOIintF	Metazoa	GGWACWGGWTGAACWGTWTAYCCYCC	(Leray et al., 2013)
	jgHCO2198	Marine invertebrates	TAIACYTCIGGRTGICCRAARAAYCA	(Geller, Meyer, Parker, & Hawk, 2013)
BF1	BF1	Freshwater macroinvertebrates	ACWGGWTGRACWGTNTAYCC	(Elbrecht & Leese, 2017)
	BR2	Freshwater macroinvertebrates	TCDGGRTGNCCRAARAAYCA	(Elbrecht & Leese, 2017)
BF2	BF2	Freshwater macroinvertebrates	GCHCCHGAYATRGCHTTYCC	(Elbrecht & Leese, 2017)
	BR2	Freshwater macroinvertebrates	TCDGGRTGNCCRAARAAYCA	(Elbrecht & Leese, 2017)
fwh1	fwhF1	Freshwater macroinvertebrates	YTCHACWAAYCAYAARGAYATYGG	(Vamos, Elbrecht, & Leese, 2017)
	fwhR1	Freshwater macroinvertebrates	ARTCARTTWCCRAAHCCHCC	(Vamos et al., 2017)

* This primer sequence was published based on its alignment to the plus strand but is shown here in the 5'-3' orientation

Table S2. Description of the datasets analyzed in Part A and Part B.

Experiment	Dataset	Proportion of dataset comprised of nuMTs (%)	Average gene length (bp)	Average nuMT length (bp)	Gene GC content (%)	nuMT GC content (%)
Artificial DNA barcoding dataset. COI genes and nuMTs from 10 species	Full length COI barcodes and nuMT sequences	19	659.6	508.1	32.0	30.8
Perturbed community dataset	Control full length COI barcodes, no nuMTs	0	615	NA	31	NA
Perturbed community dataset	Full length COI barcodes, nuMTs introduced through point mutations to decrease GC content	19	615	615	31	29
Perturbed community dataset	Full length COI barcode, nuMTs introduced through frameshift mutations (indels)	19	615	607	31	31
Perturbed community dataset	Control short COI barcode sequences, no nuMTs	0	307** - 308*	NA	30*-32**	NA
Perturbed community dataset	Short COI barcode sequences, nuMTs with decreased GC content	19	307** - 308*	308	30*-32**	28-29
Perturbed community dataset	Short COI barcode sequences, nuMTs with indels	19	307** - 308*	304	30*-32**	31-32
Perturbed community dataset	Control full length COI barcode	0	622	NA	31	NA

	sequences, no nuMTs					
Perturbed community dataset	Full length COI barcodes, twice the number of nuMTs with decreased GC content	38	622	622	31	28
Perturbed community dataset	Full length COI barcodes, twice the number of nuMTs with indels	38	622	614	31	32
Perturbed community dataset	Control full length COI barcode sequences, no nuMTs	0	622	NA	31	NA
Perturbed community dataset	Full length COI barcodes, half the number of nuMTs with decreased GC content	9.5	622	623	31	28
Perturbed community dataset	Full length COI barcodes, half the number of nuMTs with indels	9.5	622	615	31	32

* 5’ fragment

** 3’ fragment

Fig S1. COI gene sequences accumulate substitutions in synonymous sites. For 10 species with annotated COI genes and nuMTs, we did a pairwise comparison of nucleotide substitutions in non-synonymous and synonymous sites: a) COI barcode sequences tend to accumulate substitutions in synonymous sites. In contrast, COI nuMTs tend to accumulate substitutions in non-synonymous sites. After filtering out pairwise comparisons between species with < 0.01 substitutions in synonymous sites (sequences too similar to yield a reliable dN/dS estimate) or > 2 substitutions in synonymous sites (sequences that have accumulated too many substitutions to yield a reliable dN/dS estimate), it was only possible to analyze dN/dS ratios for COI barcode sequences. b) Most pairwise comparisons of COI gene sequences resulted in dN/dS ratios < 1 consistent with purifying selection pressure and the conservation of a protein sequence.

a)

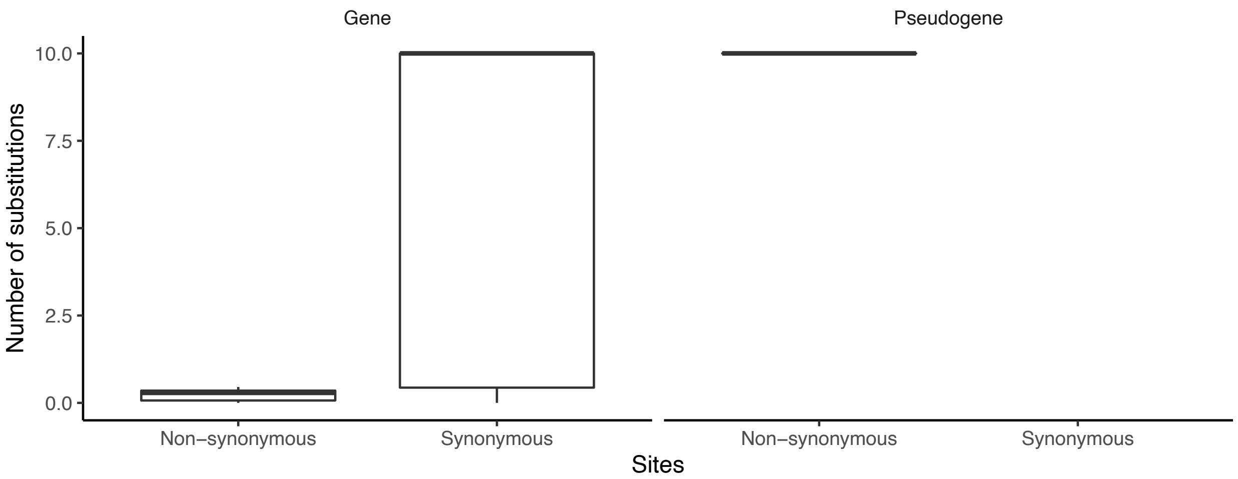

Gene
Pseudogene
10.0
7.5
5.0
2.5
0.0
Number of substitutions
Non−synonymous
Synonymous
Non−synonymous
Synonymous
Sites

b)

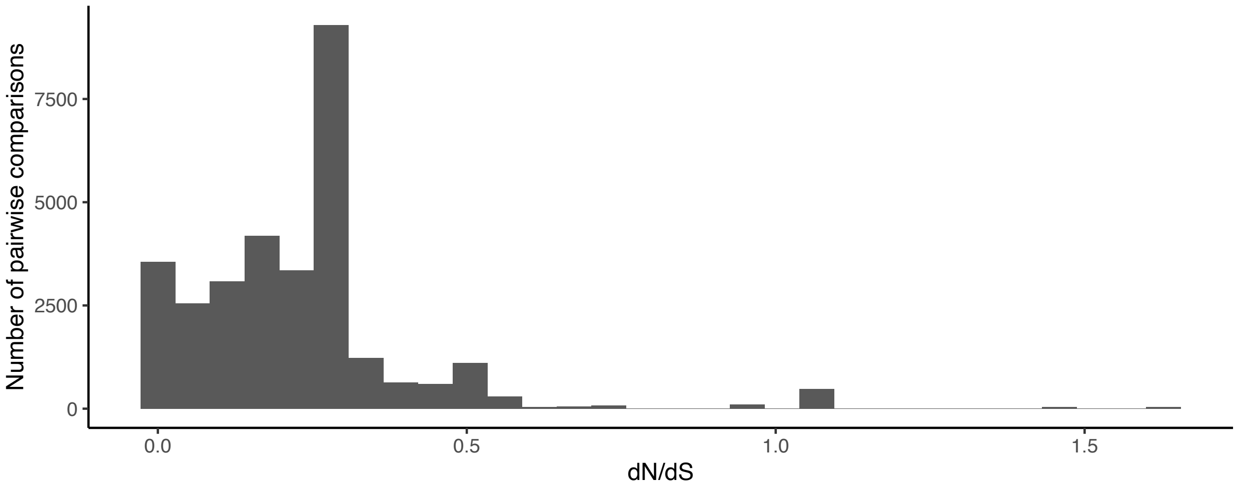

7500
5000
2500
0
Number of pairwise comparisons
0.0
0.5
1.0
1.5
dN/dS

Fig S2. ***Bemisia tabaci* COI pseudogenes cluster together on long branches.** A mid-point rooted neighbor joining phylogram using the Kimura 2-parameter model of nucleotide substitution included gene and known pseudogene sequences. Sequences annotated in GenBank as a nuclear copy of a mitochondrial gene are shown in red. Nodes with greater than 70% bootstrap support are labelled.

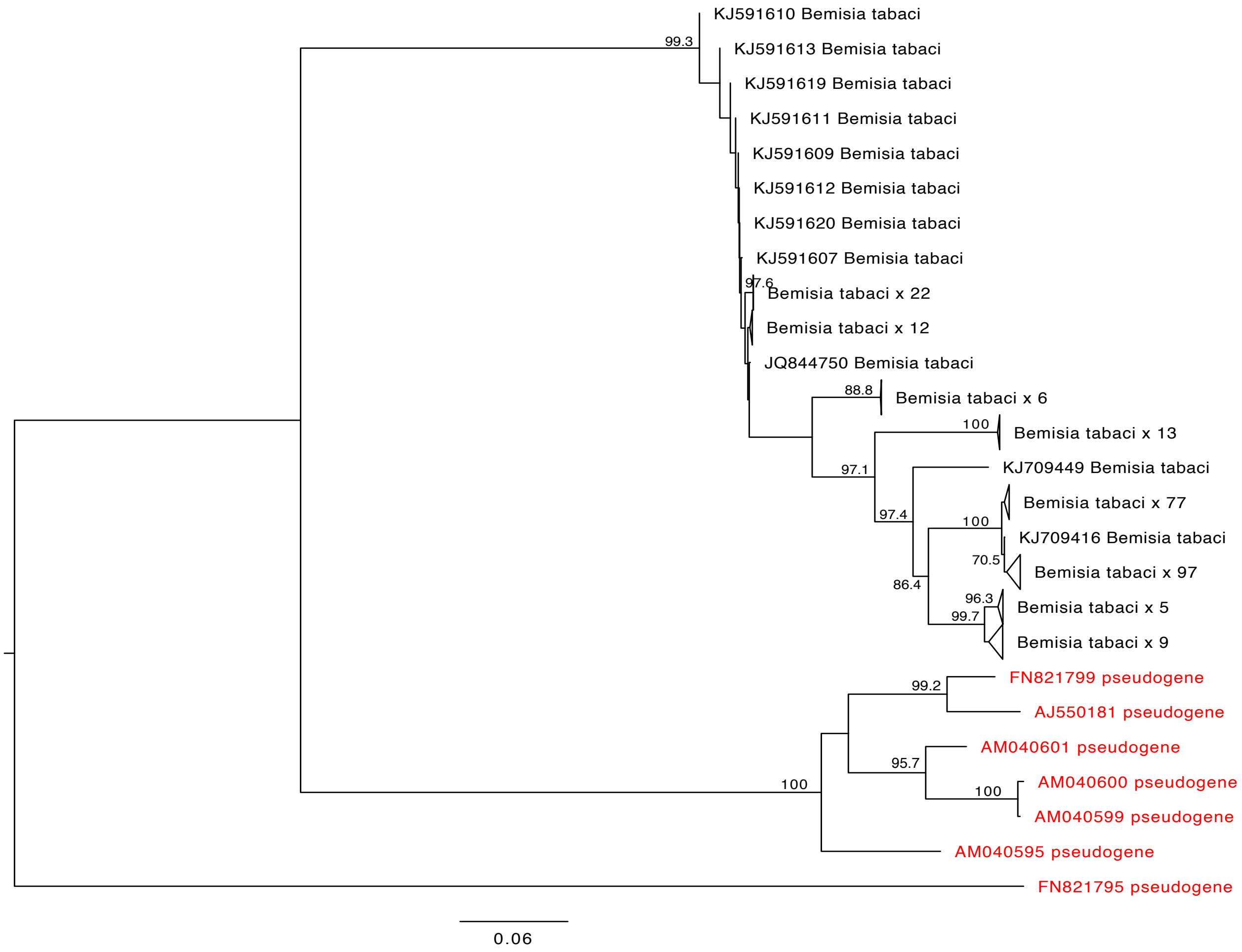

Fig S3. A single *Xylosandrus germanus* COI pseudogene sequence is found on a long branch. A mid-point rooted neighbor joining phylogram using the Kimura 2-parameter model of nucleotide substitution included COI gene sequences as well as a sequence annotated in GenBank as a nuclear copy of a mitochondrial gene (red). Nodes with greater than 70% bootstrap support are labelled.

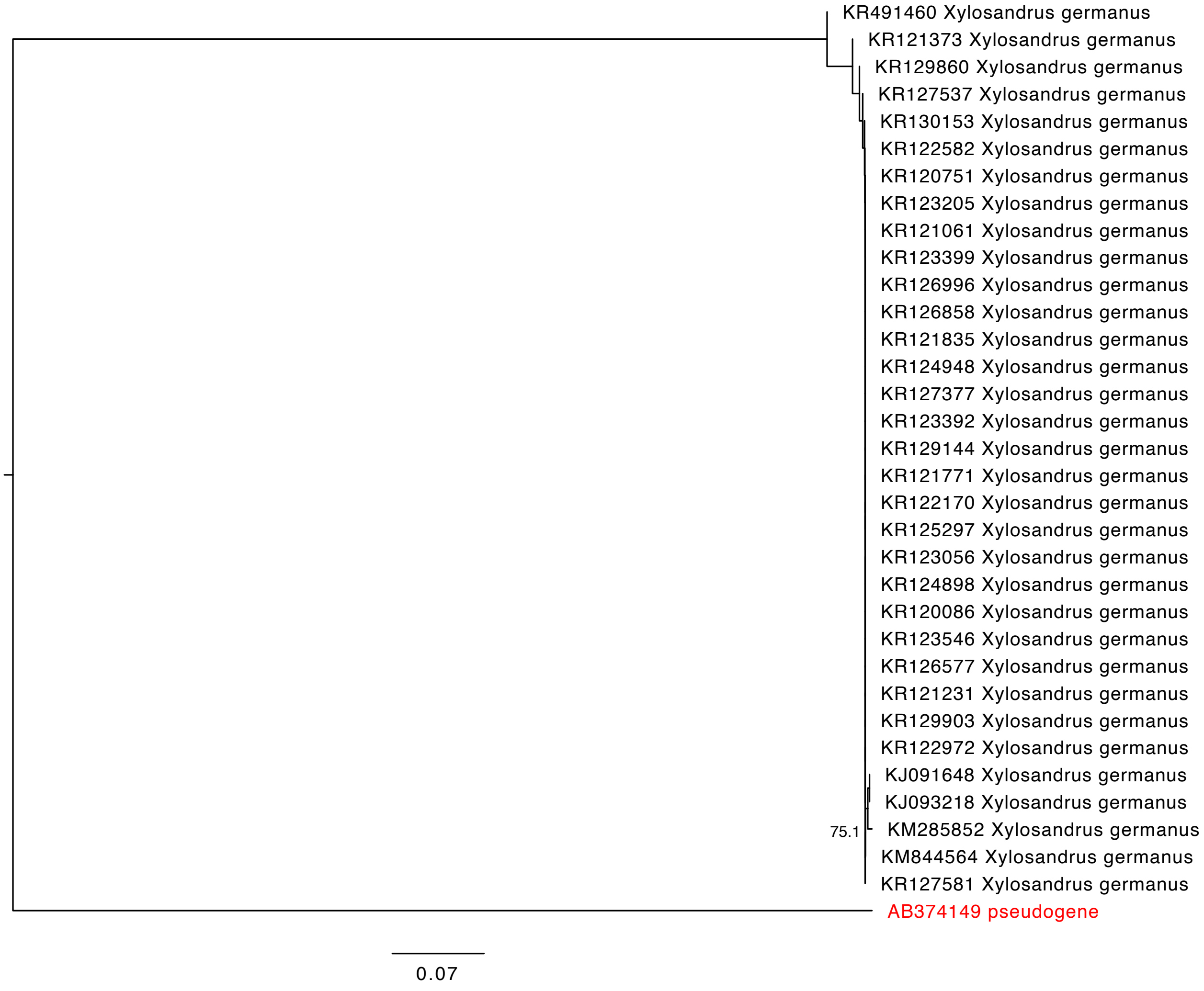

Fig S4. A single *Triatoma dimidiata* COI pseudogene sequence is found on a long branch. A mid-point rooted neighbor joining phylogram using the Kimura 2-parameter model of nucleotide substitution included COI gene sequences as well as a sequence annotated in GenBank as a nuclear copy of a mitochondrial gene (red). Nodes with greater than 70% bootstrap support are labelled.

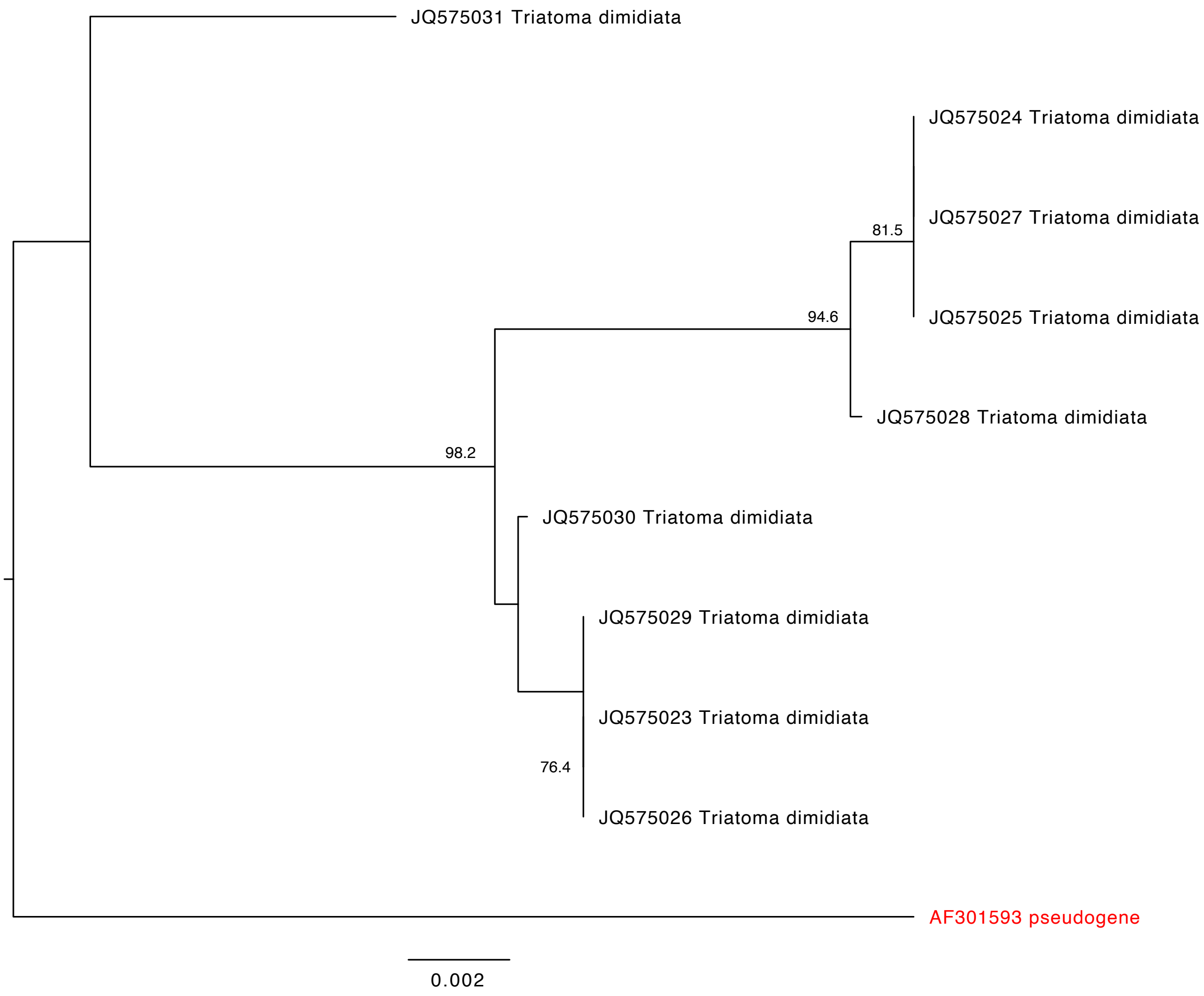

Fig S5. A single *Trialeurodes vaporariorum* COI pseudogene sequence is found on a long branch. A mid-point rooted neighbor joining phylogram using the Kimura 2-parameter model of nucleotide substitution included COI gene sequences as well as a sequence annotated in GenBank as a nuclear copy of a mitochondrial gene (red). Nodes with greater than 70% bootstrap support are labelled.

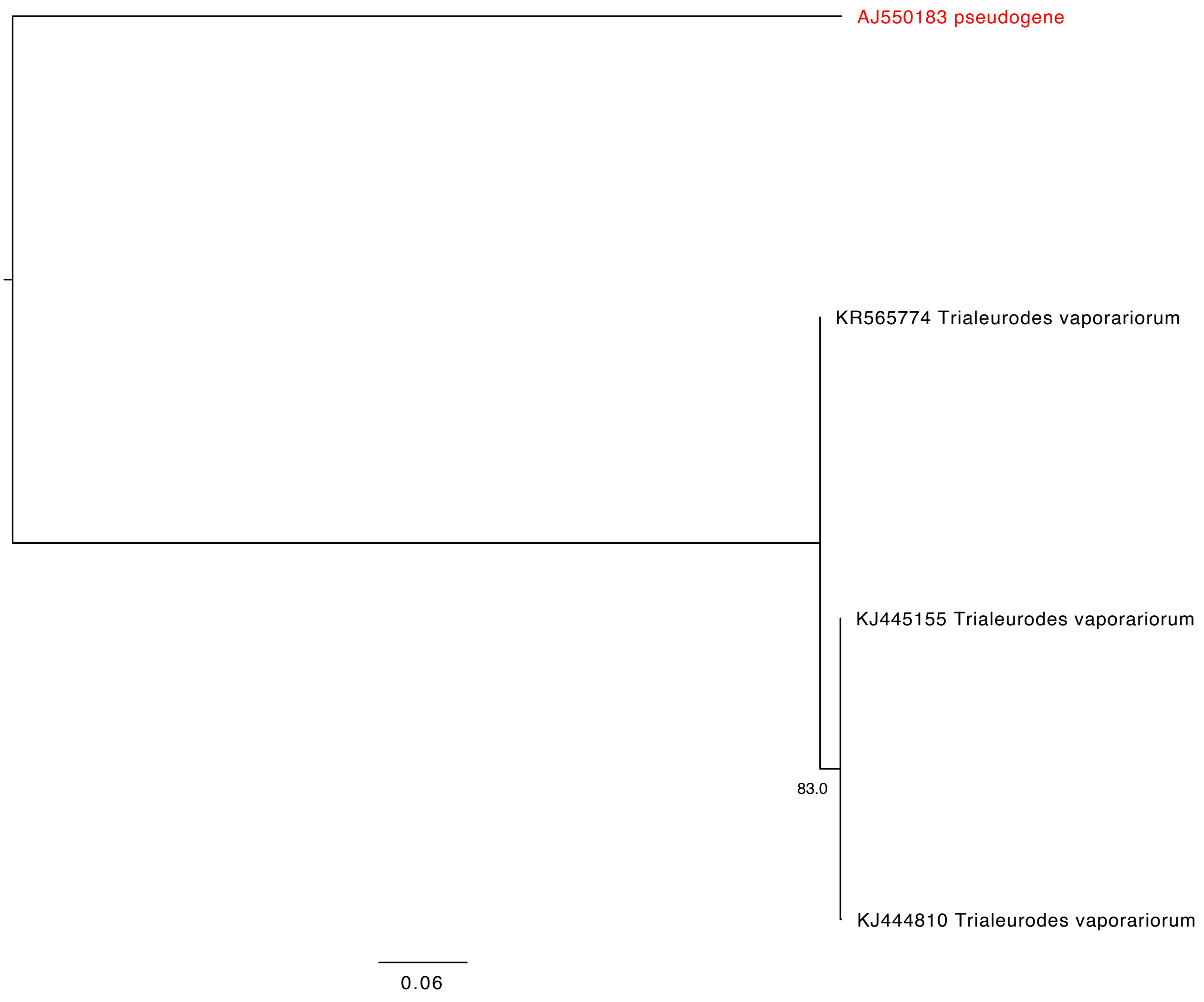

Fig S6. *Melissotarsus insularis* COI gene and annotated pseudogene sequences are often found in intermixed clusters. A mid-point rooted neighbor joining phylogram using the Kimura 2-parameter model of nucleotide substitution included COI gene sequences as well as sequences annotated in GenBank as a nuclear copy of a mitochondrial gene (red). Nodes with greater than 70% bootstrap support are labelled. Clusters of nearly identical sequences were collapsed.

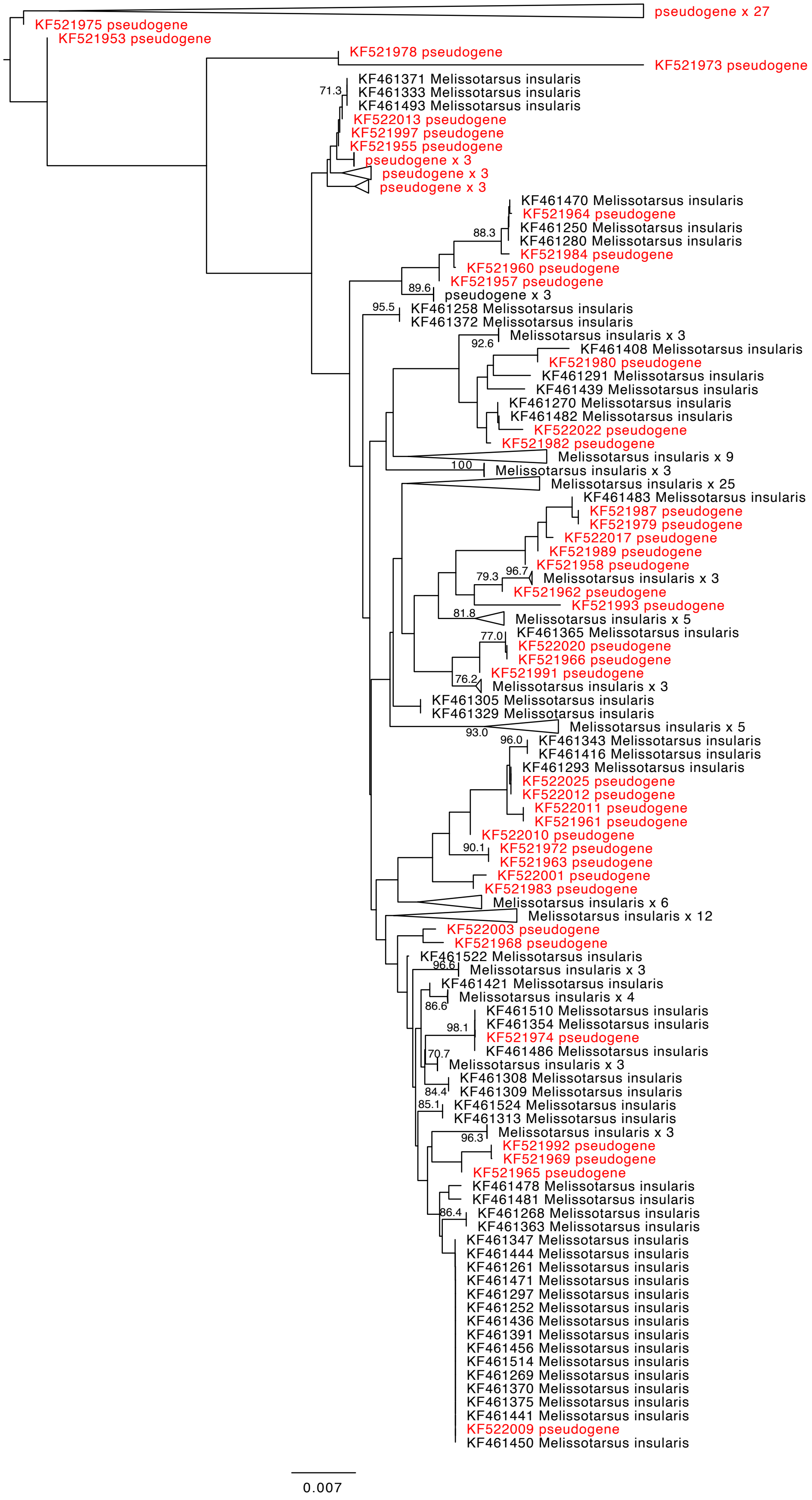
pseudogene x 27
KF521975 pseudogene
KF521953 pseudogene
KF521978 pseudogene
KF521973 pseudogene
71.3
KF461371 Melissotarsus insularis
KF461333 Melissotarsus insularis
KF461493 Melissotarsus insularis
KF522013 pseudogene
KF521997 pseudogene
KF521955 pseudogene
pseudogene x 3
pseudogene x 3
pseudogene x 3
KF461470 Melissotarsus insularis
KF521964 pseudogene
88.3
KF461250 Melissotarsus insularis
KF461280 Melissotarsus insularis
KF521984 pseudogene
KF521960 pseudogene
KF521957 pseudogene
89.6
pseudogene x 3
95.5
KF461258 Melissotarsus insularis
KF461372 Melissotarsus insularis
Melissotarsus insularis x 3
92.6
KF461408 Melissotarsus insularis
KF521980 pseudogene
KF461291 Melissotarsus insularis
KF461439 Melissotarsus insularis
KF461270 Melissotarsus insularis
KF461482 Melissotarsus insularis
KF522022 pseudogene
KF521982 pseudogene
Melissotarsus insularis x 9
100
Melissotarsus insularis x 3
Melissotarsus insularis x 25
KF461483 Melissotarsus insularis
KF521987 pseudogene
KF521979 pseudogene
KF522017 pseudogene
KF521989 pseudogene
KF521958 pseudogene
79.3
96.7
Melissotarsus insularis x 3
KF521962 pseudogene
KF521993 pseudogene
81.8
Melissotarsus insularis x 5
KF461365 Melissotarsus insularis
77.0
KF522020 pseudogene
KF521966 pseudogene
KF521991 pseudogene
76.2
Melissotarsus insularis x 3
KF461305 Melissotarsus insularis
KF461329 Melissotarsus insularis
Melissotarsus insularis x 5
93.0
96.0
KF461343 Melissotarsus insularis
KF461416 Melissotarsus insularis
KF461293 Melissotarsus insularis
KF522025 pseudogene
KF522012 pseudogene
KF522011 pseudogene
KF521961 pseudogene
KF522010 pseudogene
90.1
KF521972 pseudogene
KF521963 pseudogene
KF522001 pseudogene
KF521983 pseudogene
Melissotarsus insularis x 6
Melissotarsus insularis x 12
KF522003 pseudogene
KF521968 pseudogene
KF461522 Melissotarsus insularis
96.6
Melissotarsus insularis x 3
KF461421 Melissotarsus insularis
Melissotarsus insularis x 4
86.6
KF461510 Melissotarsus insularis
98.1
KF461354 Melissotarsus insularis
KF521974 pseudogene
KF461486 Melissotarsus insularis
70.7
Melissotarsus insularis x 3
KF461308 Melissotarsus insularis
84.4
KF461309 Melissotarsus insularis
85.1
KF461524 Melissotarsus insularis
KF461313 Melissotarsus insularis
Melissotarsus insularis x 3
96.3
KF521992 pseudogene
KF521969 pseudogene
KF521965 pseudogene
KF461478 Melissotarsus insularis
KF461481 Melissotarsus insularis
86.4
KF461268 Melissotarsus insularis
KF461363 Melissotarsus insularis
KF461347 Melissotarsus insularis
KF461444 Melissotarsus insularis
KF461261 Melissotarsus insularis
KF461471 Melissotarsus insularis
KF461297 Melissotarsus insularis
KF461252 Melissotarsus insularis
KF461436 Melissotarsus insularis
KF461391 Melissotarsus insularis
KF461456 Melissotarsus insularis
KF461514 Melissotarsus insularis
KF461269 Melissotarsus insularis
KF461370 Melissotarsus insularis
KF461375 Melissotarsus insularis
KF461441 Melissotarsus insularis
KF522009 pseudogene
KF461450 Melissotarsus insularis
0.007

Fig S7. A single *Lepidocyrtus cyaneus* COI pseudogene sequence clusters with other gene sequences. A mid-point rooted neighbor joining phylogram using the Kimura 2-parameter model of nucleotide substitution included COI gene sequences as well as a sequence annotated in GenBank as a nuclear copy of a mitochondrial gene (red). Nodes with greater than 70% bootstrap support are labelled.

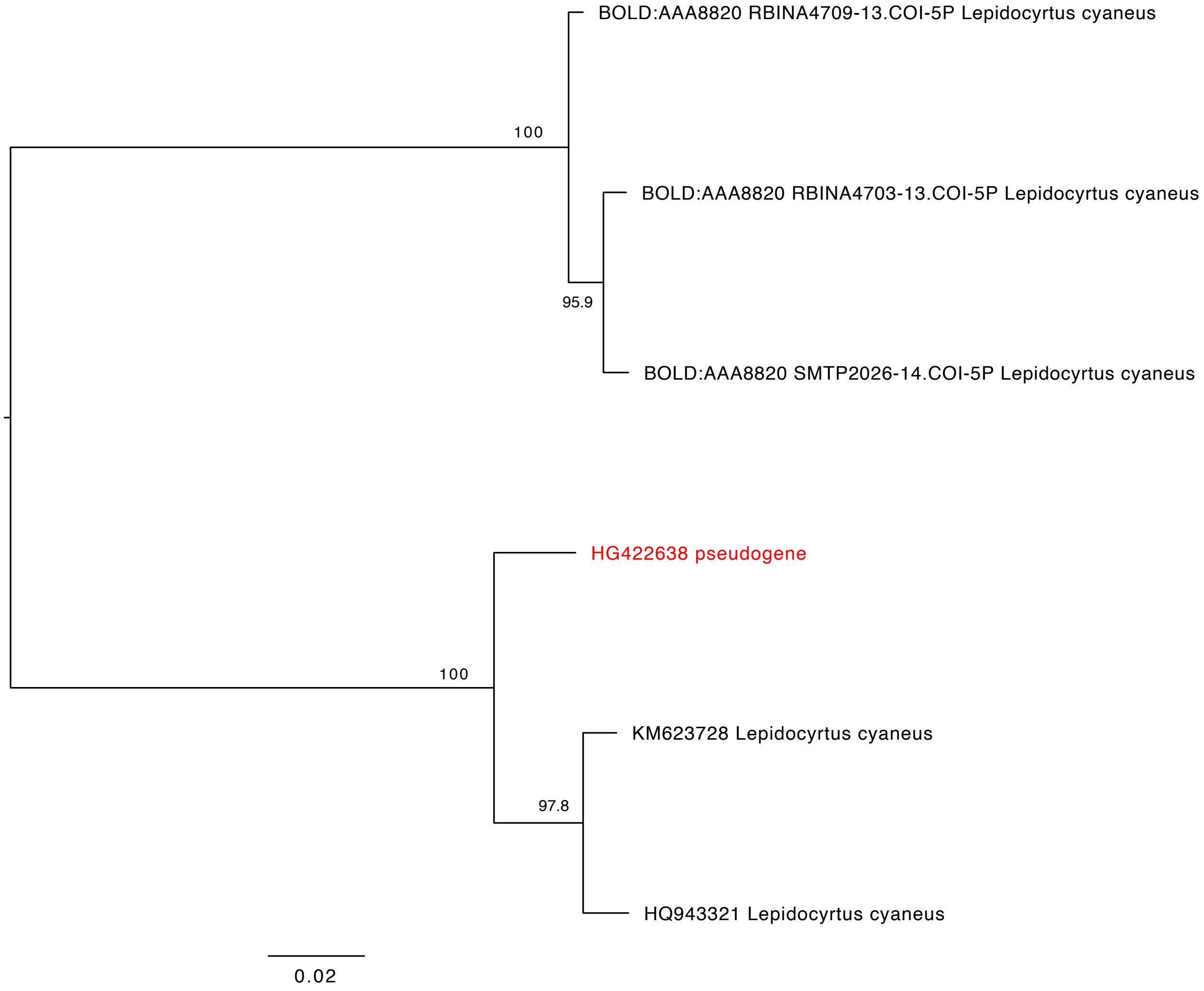

Fig S8. Two *Halictus rubicundus* COI pseudogene sequences cluster together near other gene sequences. A mid-point rooted neighbor joining phylogram using the Kimura 2-parameter model of nucleotide substitution included COI gene sequences as well as two sequences annotated in GenBank as a nuclear copy of a mitochondrial gene (red). Nodes with greater than 70% bootstrap support are labelled.

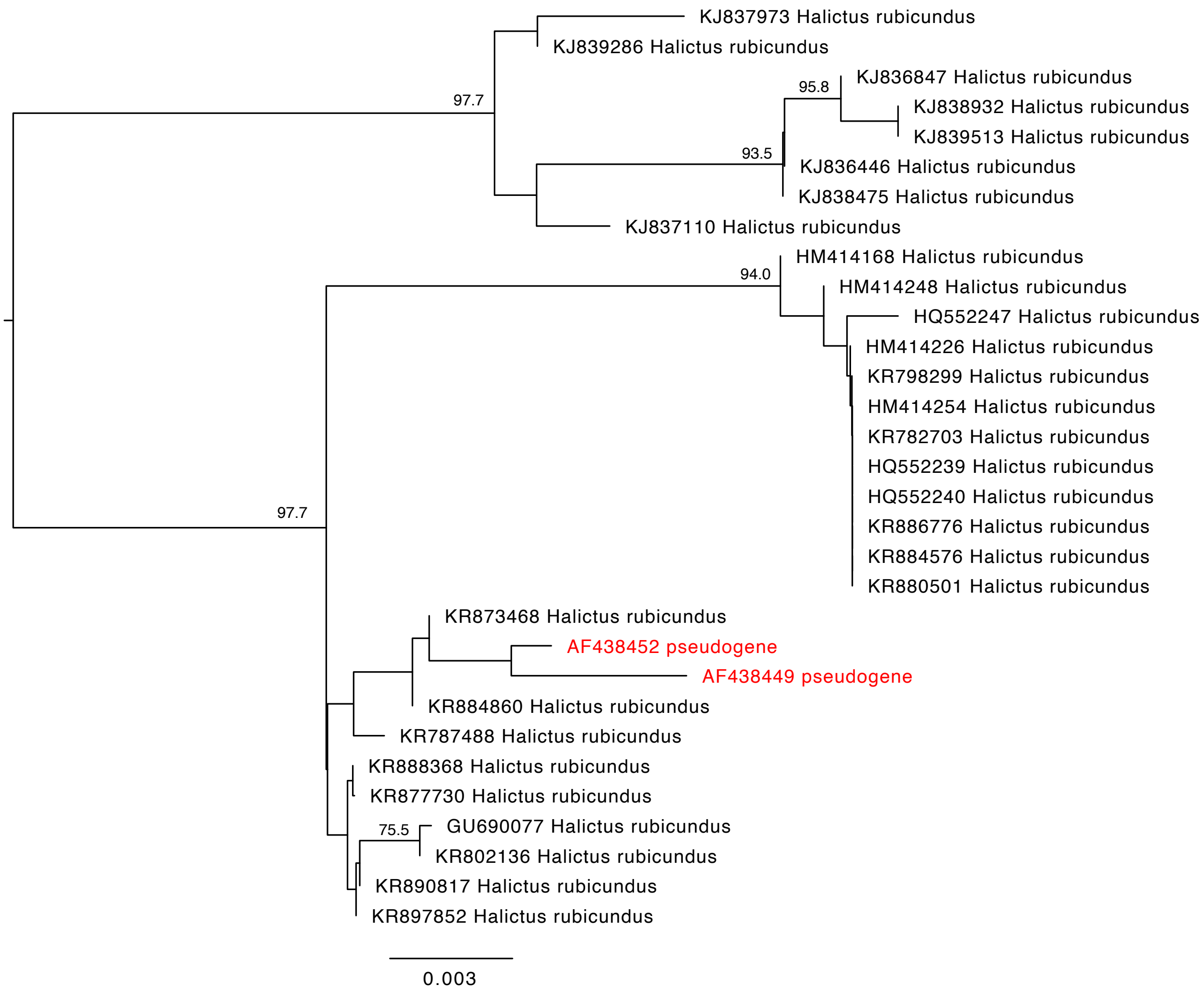

Fig S9. Several *Goneplax rhomboides* COI pseudogene sequences cluster together. A mid-point rooted neighbor joining phylogram using the Kimura 2-parameter model of nucleotide substitution included COI gene sequences as well as sequences annotated in GenBank as a nuclear copy of a mitochondrial gene (red). Nodes with greater than 70% bootstrap support are labelled.

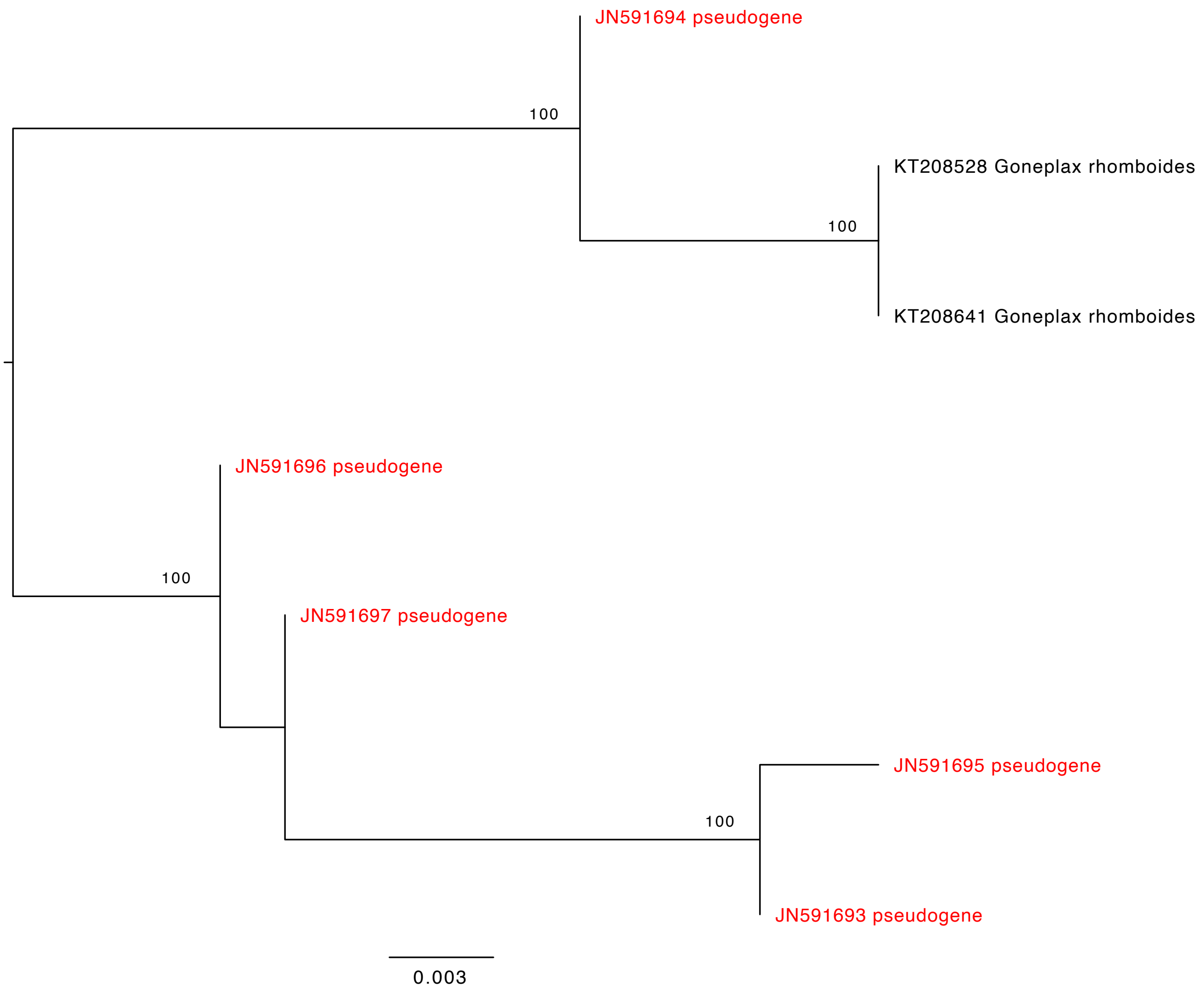

Fig S10. A single *Ectatomma gibbum* COI pseudogene sequence is found on its own branch. A mid-point rooted neighbor joining phylogram using the Kimura 2-parameter model of nucleotide substitution included COI gene sequences as well as a sequence annotated in GenBank as a nuclear copy of a mitochondrial gene (red). Nodes with greater than 70% bootstrap support are labelled.

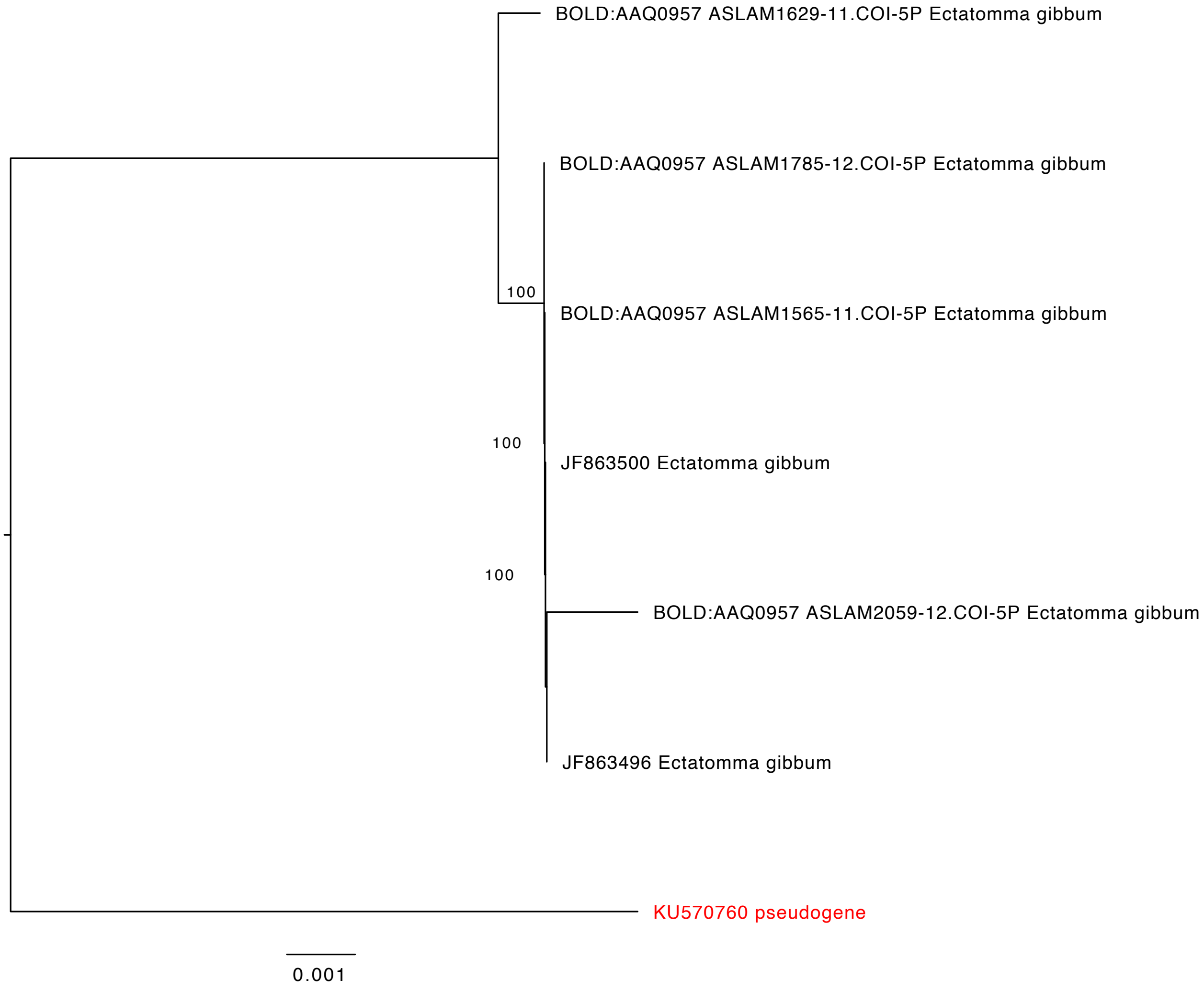

Fig S11. *Cyphoderris monstrosa* COI gene and annotated pseudogene sequences sometimes cluster with regular gene sequences. A mid-point rooted neighbor joining phylogram using the Kimura 2-parameter model of nucleotide substitution included COI gene sequences as well sequences annotated in GenBank as a nuclear copy of a mitochondrial gene (red). Nodes with greater than 70% bootstrap support are labelled.

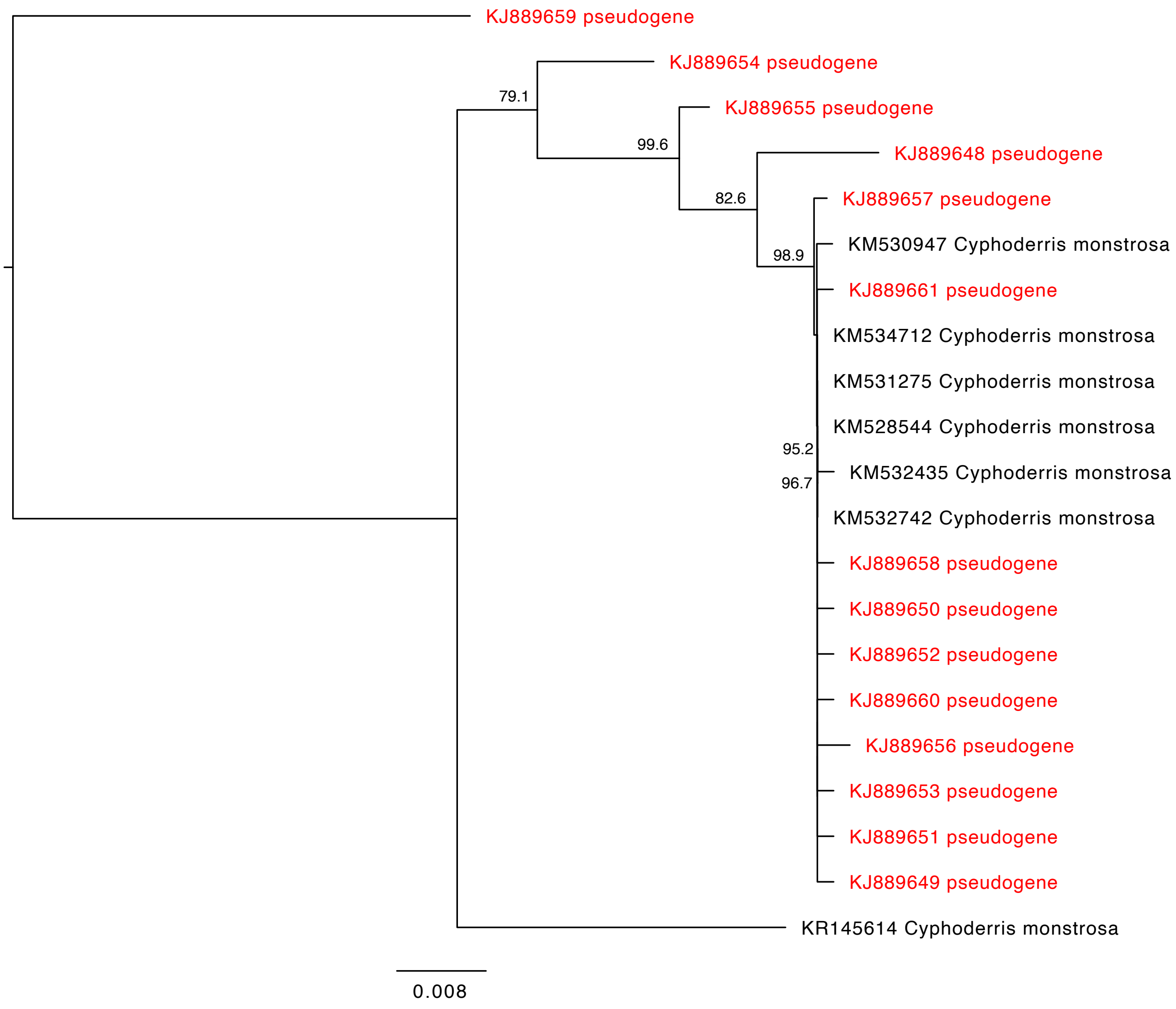

Fig S12. Sensitivity and specificity were used to assess the effectiveness of our two pseudogene filtering approaches. The vertical dashed line represents a threshold used to delimit nuMT sequences. The ability to detect pseudogenes represents the positive condition. Correctly removed nuMTs are true positives (TP). Incorrectly filtered COI gene sequences (genes) represents false positives (FP). Correctly retained genes represents true negatives (TN). Incorrectly retained nuMTs represents false negatives (FN).

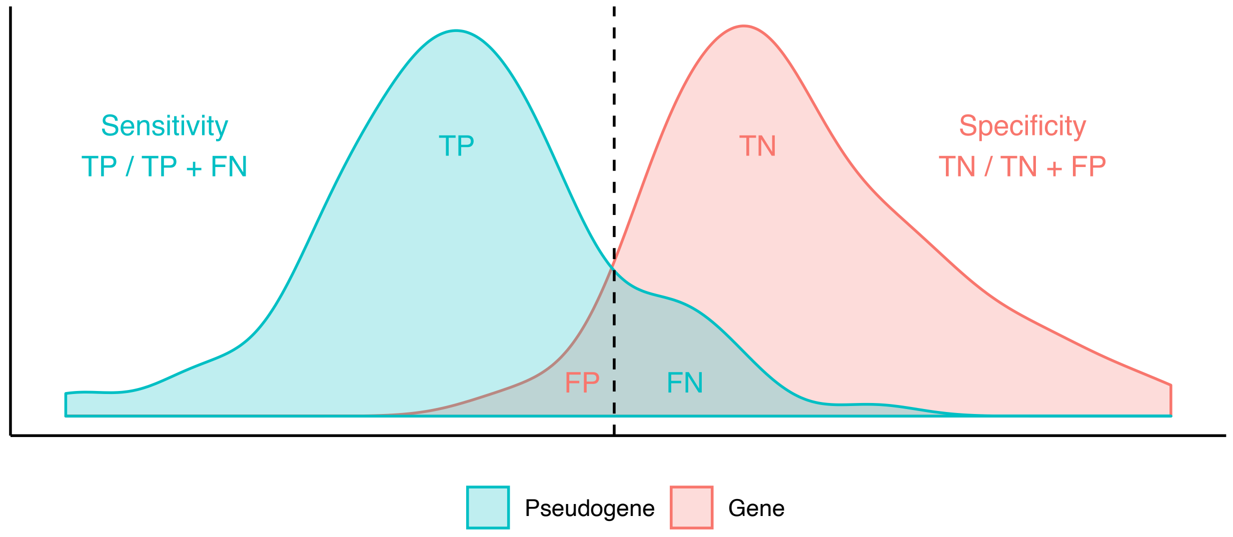

Fig S13. Halving COI sequence lengths results in fewer pseudogenes removed compared with full length COI barcode sequences. Each column shows the results from a particular simulation: a controlled community with nuMTs absent, a community with simulated nuMTs with a reduced GC content, and a community with simulated nuMTs with frameshift mutations (introduced indels). The top two panels show the length variation of sequences in the longest retained open reading frame for short sequences sampled from the 5' and 3' end of COI barcode sequences. The solid vertical line indicates half the length of a typical COI barcode at 329 bp. The two vertical dashed lines shows the boundaries for identifying ORFs with outlier lengths. The bottom two panels show the nucleotide bit score for short sequences sampled from the 5' and 3' ends of COI barcode sequences. The dashed vertical line shows the boundary for identifying sequences with unusually short scores.

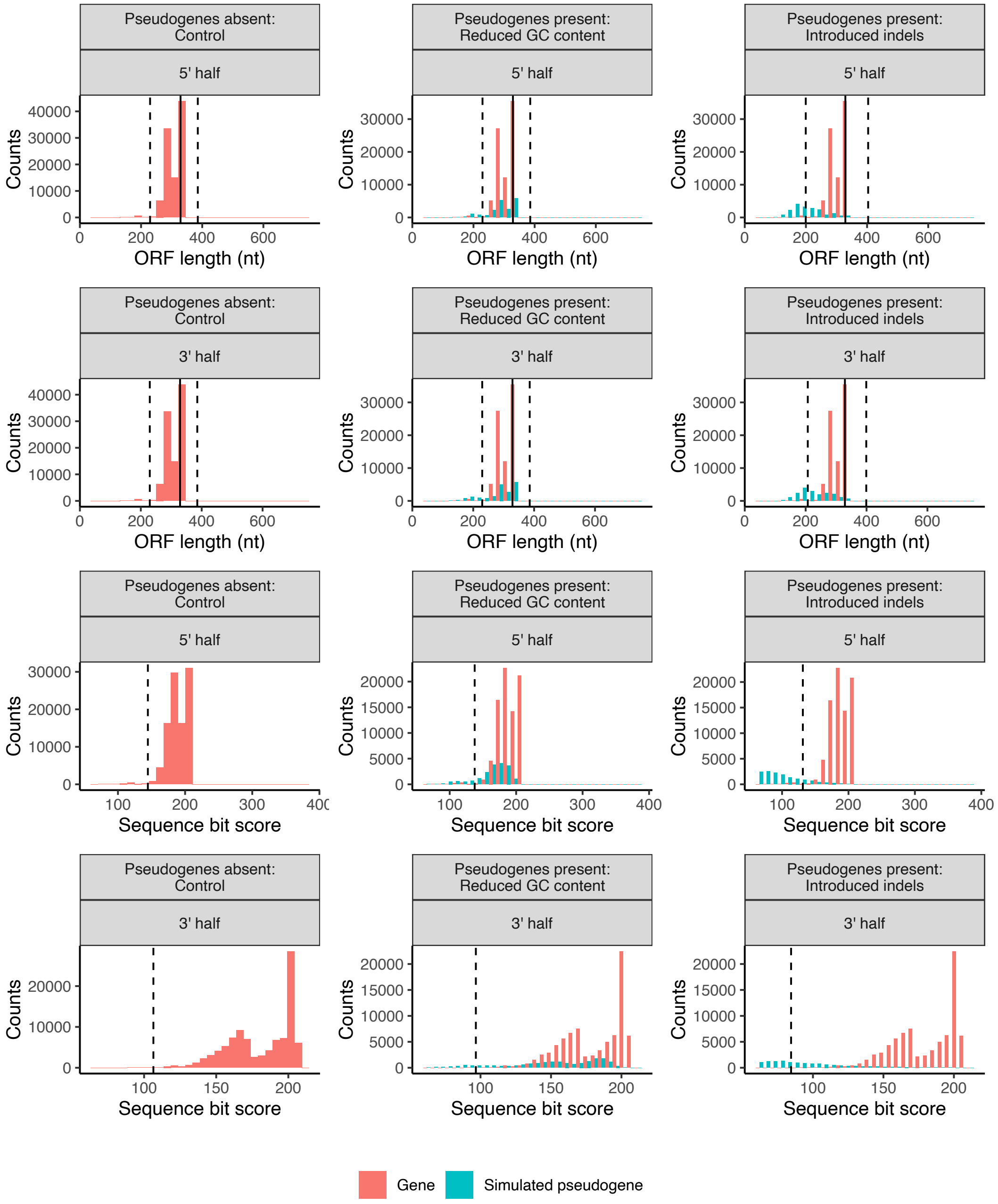
Pseudogenes absent: Control
Pseudogenes present: Reduced GC content
Pseudogenes present: Introduced indels
5' half
3' half
Counts
ORF length (nt)
Sequence bit score
Gene
Simulated pseudogene

Fig S14. Doubling the proportion of mutated sequences greatly reduces the number of pseudogenes removed. Each column shows the results from a particular simulation: a controlled community with nuMTs absent, a community with nuMTs that have a reduced GC content, and a community with nuMTs with frameshift mutations (introduced indels). The top panel shows the length variation of sequences in the longest retained open reading frame. The solid vertical line indicates the length of a typical COI barcode at 658 bp. The two vertical dashed lines shows the boundaries for identifying ORFs with outlier lengths. The bottom panel shows the sequence bit score variation. The vertical dashed line shows the boundary for identifying sequences with small outlier scores.

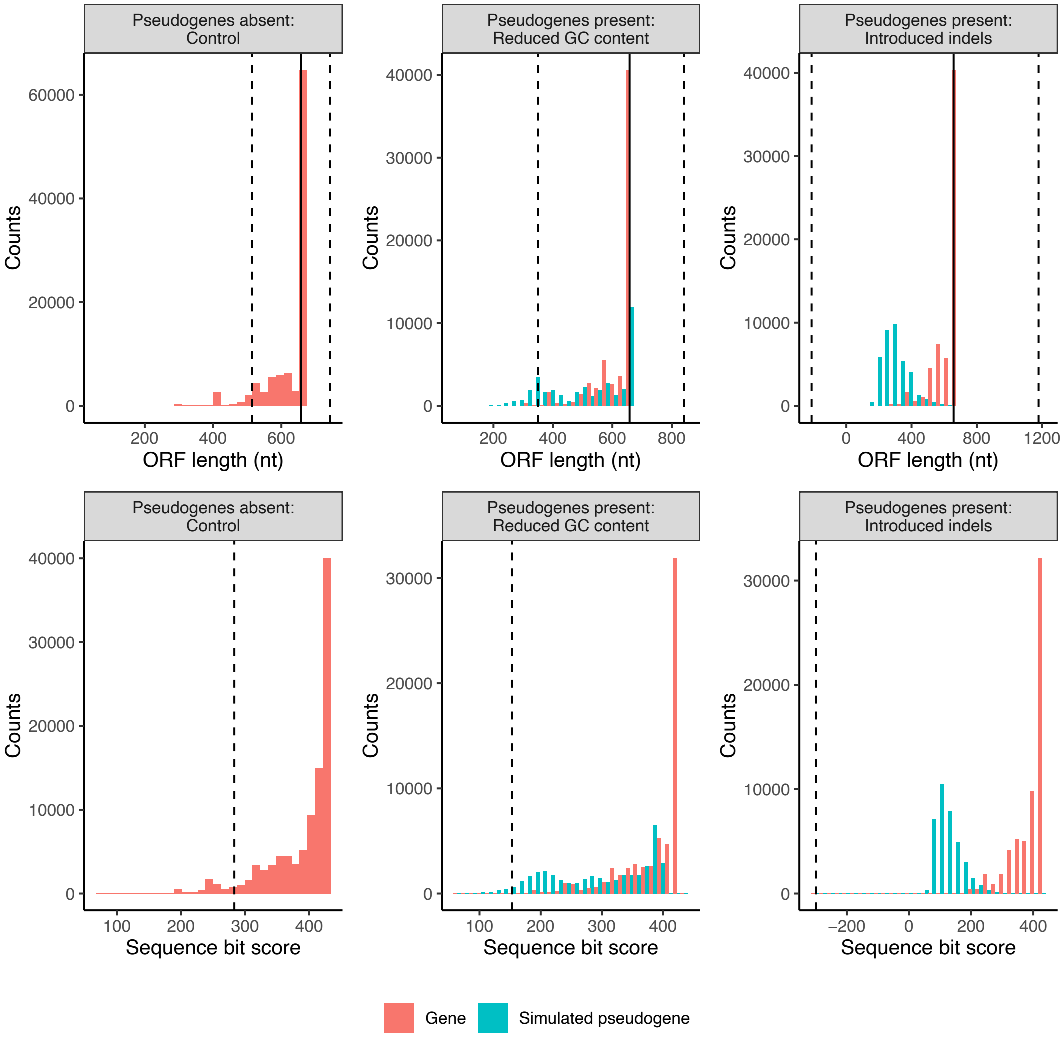

Pseudogenes absent: Control
Pseudogenes present: Reduced GC content
Pseudogenes present: Introduced indels
Counts
ORF length (nt)
Sequence bit score
Gene
Simulated pseudogene

Fig S15. Halving the proportion of mutated sequences increases the number of pseudogenes removed. Each column shows the results from a particular simulation: a controlled community with nuMTs absent, a community with nuMTs that have a reduced GC content, and a community with nuMTs with frameshift mutations (introduced indels). The top panel shows the length variation of sequences in the longest retained open reading frame. The solid vertical line indicates the length of a typical COI barcode at 658 bp. The two vertical dashed lines shows the boundaries for identifying ORFs with outlier lengths. The bottom panel shows the sequence bit score variation. The vertical dashed line shows the boundaries for identifying sequences with short outliers scores.

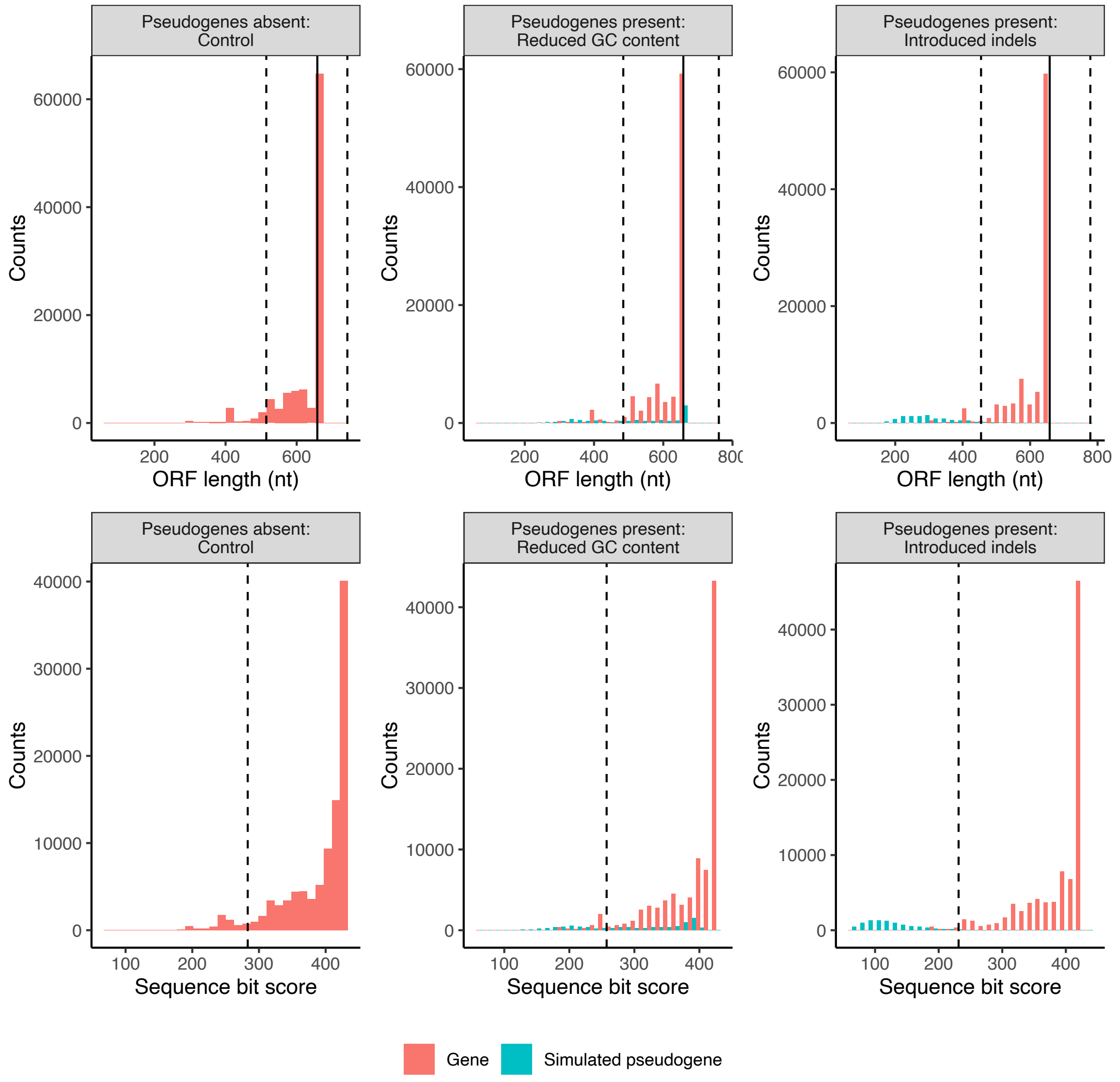
Pseudogenes absent: Control
Pseudogenes present: Reduced GC content
Pseudogenes present: Introduced indels
Counts
ORF length (nt)
Sequence bit score
Gene
Simulated pseudogene

References

Elbrecht, V., & Leese, F. (2017). Validation and Development of COI Metabarcoding Primers for Freshwater Macroinvertebrate Bioassessment. *Frontiers in Environmental Science*, *5*, 11. doi: 10.3389/fenvs.2017.00011

Folmer, O., Black, M., Hoeh, W., Lutz, R., & Vrijenhoek, R. (1994). DNA primers for amplification of mitochondrial cytochrome c oxidase subunit I from diverse metazoan invertebrates. *Molecular Marine Biology and Biotechnology*, *3*(5), 294–299.

Geller, J., Meyer, C., Parker, M., & Hawk, H. (2013). Redesign of PCR primers for mitochondrial cytochrome c oxidase subunit I for marine invertebrates and application in all-taxa biotic surveys. *Molecular Ecology Resources*, *13*(5), 851–861. doi: 10.1111/1755-0998.12138

Gibson, J., Shokralla, S., Curry, C., Baird, D. J., Monk, W. A., King, I., & Hajibabaei, M. (2015). Large-Scale Biomonitoring of Remote and Threatened Ecosystems via High-Throughput Sequencing. *PLOS ONE*, *10*(10), e0138432. doi: 10.1371/journal.pone.0138432

Gibson, J., Shokralla, S., Porter, T. M., King, I., Konynenburg, S. van, Janzen, D. H., … Hajibabaei, M. (2014). Simultaneous assessment of the macrobiome and microbiome in a bulk sample of tropical arthropods through DNA metasystematics. *Proceedings of the National Academy of Sciences*, *111*(22), 8007–8012. doi: 10.1073/pnas.1406468111

Hajibabaei, M., Spall, J. L., Shokralla, S., & van Konynenburg, S. (2012). Assessing biodiversity of a freshwater benthic macroinvertebrate community through non-destructive environmental barcoding of DNA from preservative ethanol. *BMC Ecology*, *12*, 28. doi: 10.1186/1472-6785-12-28

Leray, M., Yang, J. Y., Meyer, C. P., Mills, S. C., Agudelo, N., Ranwez, V., … Machida, R. J. (2013). A new versatile primer set targeting a short fragment of the mitochondrial COI region for metabarcoding metazoan diversity: Application for characterizing coral reef fish gut contents. *Frontiers in Zoology*, *10*(1), 34. doi: 10.1186/1742-9994-10-34

Vamos, E., Elbrecht, V., & Leese, F. (2017). Short COI markers for freshwater macroinvertebrate metabarcoding. *Metabarcoding and Metagenomics*, *1*, e14625. doi: 10.3897/mbmg.1.14625
